# Left-Right Side-Specific Neuropeptide Mechanism Mediates Contralateral Responses to a Unilateral Brain Injury

**DOI:** 10.1523/ENEURO.0548-20.2021

**Published:** 2021-05-22

**Authors:** Hiroyuki Watanabe, Olga Nosova, Daniil Sarkisyan, Marlene Storm Andersen, Liliana Carvalho, Vladimir Galatenko, Igor Bazov, Nikolay Lukoyanov, Gisela H. Maia, Mathias Hallberg, Mengliang Zhang, Jens Schouenborg, Georgy Bakalkin

**Affiliations:** 1 Department of Pharmaceutical Biosciences, Uppsala University, Uppsala, Sweden, 751 24; 2 Department of Molecular Medicine, University of Southern Denmark, Odense, Denmark, 5230; 3 Departamento de Biomedicina da Faculdade de Medicina da Universidade do Porto, Instituto de Investigação e Inovação em Saúde, Instituto de Biologia Molecular e Celular, Porto, Portugal, 4200-135; 4 Faculty of Mechanics and Mathematics, Lomonosov Moscow State University, Moscow, Russia, 119991; 5 Medibrain, Vila do Conde, Porto, Portugal, 4480-807; 6 Brain Research Institute, Porto, Portugal, 4200-135; 7 Neuronano Research Center, Department of Experimental Medical Science, Lund University, Lund, Sweden, 223 81

**Keywords:** brain injury, postural asymmetry, withdrawal reflexes, opioid system, left-right side-specific regulation

## Abstract

Neuropeptides are implicated in control of lateralized processes in the brain. A unilateral brain injury (UBI) causes the contralesional sensorimotor deficits. To examine whether opioid neuropeptides mediate UBI induced asymmetric processes we compared effects of opioid antagonists on the contralesional and ipsilesional hindlimb responses to the left-sided and right-sided injury in rats. UBI induced hindlimb postural asymmetry (HL-PA) with the contralesional hindlimb flexion, and activated contralesional withdrawal reflex of extensor digitorum longus (EDL) evoked by electrical stimulation and recorded with EMG technique. No effects on the interossei (Int) and peroneaus longus (PL) were evident. The general opioid antagonist naloxone blocked postural effects, did not change EDL asymmetry while uncovered cryptic asymmetry in the PL and Int reflexes induced by UBI. Thus, the spinal opioid system may either mediate or counteract the injury effects. Strikingly, effects of selective opioid antagonists were the injury side-specific. The μ-antagonist β-funaltrexamine (FNA) and κ-antagonist nor-binaltorphimine (BNI) reduced postural asymmetry after the right but not left UBI. In contrast, the δ-antagonist naltrindole (NTI) inhibited HL-PA after the left but not right-side brain injury. The opioid gene expression and opioid peptides were lateralized in the lumbar spinal cord, and coordination between expression of the opioid and neuroplasticity-related genes was impaired by UBI that together may underlie the side-specific effects of the antagonists. We suggest that mirror-symmetric neural circuits that mediate effects of left and right brain injury on the contralesional hindlimbs are differentially controlled by the lateralized opioid system.

## Visual Abstract

**Figure:**
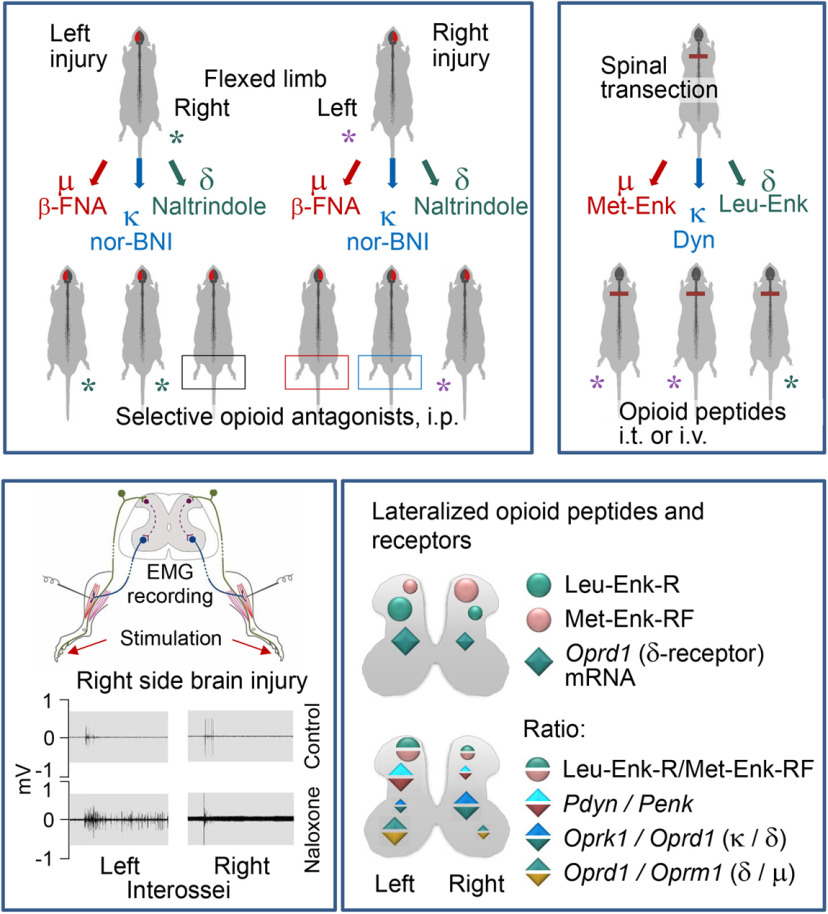

## Significance Statement

Functional specialization of the left and right hemispheres is an organizing principle of the brain. Lasting regulation of lateralized processes may be accomplished by paracrine neurohormonal mechanisms that preferentially operate in the left or right hemisphere. Our findings support this hypothesis by demonstration that mirror-symmetric neural circuits that control the left and right hindlimbs may be regulated by the left-side and right-side specific neuropeptide mechanisms. Neuropeptides may differentially target the left and right counterparts of these circuits, and in this way control the left-right balance in their functional performance. This bipartite mechanism may be based on lateralization of the neuropeptide systems, and may operate in the spinal cord or control neural pathways descending from the brain to contralateral motoneurons.

## Introduction

External symmetry and functional specialization of the left and right hemispheres are a fundamental principle of nervous system organization in bilaterians (Craig, 2005; MacNeilage et al., 2009; Concha et al., 2012; Duboc et al., 2015; Güntürkün and Ocklenburg, 2017). Language, spatial cognition, motor functions, emotions, and pain are lateralized. Asymmetries of neural circuits of the left and right hemisphere subserving these functions may be a basis for the lateralization of these functions (Concha et al., 2012; Gotts et al., 2013; Caeyenberghs and Leemans, 2014; Horstick et al., 2020).

Several studies suggest that the lateralized neural circuits are controlled by local paracrine regulators, including neuropeptides and neurotransmitters that operate either on the left or right side (Deliagina et al., 2000; Kawakami et al., 2003; Zink et al., 2011; Hussain et al., 2012; Marlin et al., 2015; Watanabe et al., 2015; Kononenko et al., 2017, 2018; Nation et al., 2018; Phelps et al., 2019). Thus, oxytocin enables retrieval behavior by enhancing responses of the left but not right auditory cortex (Marlin et al., 2015), while the dynorphin–κ-opioid system controls processing of pain in the right but not left amygdala (Nation et al., 2018; Phelps et al., 2019). Intriguing questions are whether there is a bipartite neurohormonal mechanism that selectively regulates the processes lateralized to the left and to the right, and whether this mechanism may have a more general role and control mirror symmetric neurocircuits by selective targeting their left and right counterparts in bilaterally symmetric animals. Such a mechanism may operate locally (e.g., within the cortex), or at several levels along the neuraxis where it can balance activity of neural pathways that convey left-right-specific signals, for example, from the cerebral hemispheres to contralateral spinal motoneurons. In this scenario, impairment of the left or right counterpart of this bipartite neurohormonal mechanism by a unilateral brain injury (UBI) may disrupt the left-right balance and lead to asymmetric functional changes.

Traumatic brain injury (TBI) and stroke in patients cause postural and sensorimotor deficits that are generally contralesional and include asymmetric posture and asymmetric exacerbated withdrawal reflexes (Dewald et al., 1999; Spaich et al., 2006, 2014; Serrao et al., 2012; Wilson et al., 2017; Jamal et al., 2018). Consistently, a unilateral injury to the hindlimb sensorimotor cortex in experimental animals induces the hindlimb postural asymmetry (HL-PA) with contralesional limb flexion and asymmetry of the nociceptive withdrawal reflexes (NWRs) with greater activity on the contralesional versus ipsilesional side (Zhang et al., 2020).

Opioid peptides and receptors in the spinal cord are involved in regulation of withdrawal reflexes, sensory processes and motor functions (Clarke et al., 1992; Faber et al., 1997; Fujibayashi et al., 1998; Jankowska and Schomburg, 1998; Steffens and Schomburg, 2011; Wang et al., 2018). Previous studies demonstrated that in animals with intact brains opioid peptides and synthetic opioids mimic the effects of a unilateral brain lesion by inducing HL-PA with flexion on the side that was determined by the type of opioid agonist administered (Bakalkin et al., 1980, 1986; Chazov et al., 1981; Bakalkin and Kobylyansky, 1989; Watanabe et al., 2020). The κ- and δ-agonists induced flexion of the left and right hindlimb, respectively.

We hypothesize that the endogenous opioid peptides may function as a bipartite neurohormonal system that selectively regulates the left and right sided processes, and, by this virtue, they may differentially control the effects of the left and right UBI on the contra-ipsilesional postural and sensorimotor hindlimb responses. To test this hypothesis, we examine whether the effects of a focal unilateral injury of the hindlimb sensorimotor cortex on the formation of HL-PA and asymmetry in hindlimb NWRs are mediated through the opioid system; whether this system is lateralized in the spinal cord; and whether opioid receptor subtypes selectively mediate the effects of the left and right side UBI on HL-PA formation. Identifying neurohormonal and neurotransmitter mechanisms that convey the left-side and right-side specific messages is important for a basic understanding of the nervous system, and development of pharmacotherapy to target asymmetric postural and sensorimotor deficits secondary to TBI and stroke.

## Materials and Methods

### Animals

Adult male Sprague Dawley rats (Taconic) weighing 200–430 g were used in the study. The animals received food and water *ad libitum*, and were kept in a 12/12 h light/dark cycle (light on from 10 A.M. to 10 P.M.) at a constant environmental temperature of 21°C (humidity: 65%) and randomly assigned to their respective experimental groups. Animal experiments ware approved by local ethical committee. Experiments were performed from 9 A.M. to 4 P.M., besides electrophysiological experiments that were conducted from 9 A.M. to ∼8 P.M. After the experiments were completed, the animals were given a lethal dose of pentobarbital.

### Brain surgery and histology

The rats were anesthetized with 3% isoflurane (Abbott) in a mixture with 65% nitrous oxide and 35% oxygen. Core temperature of the animals was controlled using a feedback-regulated heating system. In the experiments involving electrophysiological recordings, an infusion of 5% glucose in Ringer acetate (pH 7.0) at a rate of 5–100 μl/min was administered via the right jugular vein, and the rats were ventilated artificially via a tracheal cannula and the expiratory CO_2_ and mean arterial blood pressure (65–140 mmHg) was monitored continuously in the right carotid artery.

The rat head was fixed in a position in which the bregma and λ were located at the same horizontal level. After local injection of lidocaine (xylocaine, 3.5 mg/ml) with adrenaline (2.2 μg/ml), the scalp was cut open and a piece of the parietal bone located 0.5–4.0 mm posterior to the bregma and 1.8–3.8 lateral to the midline (Paxinos and Watson, 2007) was removed. The part of the cerebral cortex located below the opening that includes the hind-limb representation area of the sensorimotor cortex was aspirated with a metallic pipette (tip diameter 0.5 mm) connected to an electrical suction machine (Craft Duo-Vec Suction unit, Rocket Medical Plc; Zhang et al., 2020). Care was taken to avoid damaging the white matter below the cortex. After the ablation, bleeding was stopped with a piece of Spongostone and the bone opening was covered with a piece of TissuDura (Baxter). For sham operations, animals underwent the same anesthesia and surgical procedures, but the cortex was not ablated.

Localization and size of cortical lesions were analyzed in rats with left side (*n* = 10) and right side (*n* = 11) UBI 6–18 d after injury. After perfusion with 4% paraformaldehyde the brain was removed and postfixed in the same fixative overnight. Then the brain was soaked in phosphate-buffered saline with 30% sucrose for 48 h, dissected into blocks which were then sliced into 50-μm sections with a freezing microtome. Every fourth section was stained with toluidine (Nissl stain), and all the stained sections across the lesion site were photographed and the rostrocaudal respective mediolateral extension as well as lesion volume were calculated.

### Analysis of HL-PA

The magnitude of postural asymmetry (MPA) and the side of the flexed limb were assessed as described elsewhere (Bakalkin and Kobylyansky, 1989; Watanabe et al., 2020; Zhang et al., 2020). Briefly, the measurements were performed under pentobarbital (60 mg/kg, i.p.) or isoflurane (1.5% isoflurane in a mixture of 65% nitrous oxide and 35% oxygen) anesthesia that yielded the same results. The level of anesthesia was characterized by a barely perceptible corneal reflex and a lack of overall muscle tone. The rat was placed in the prone position on the 1-mm grid paper, and the hip and knee joints were straightened by gently pulling the hindlimbs backwards for 1 cm to reach the same level. Then, the hindlimbs were set free and the MPA was measured in millimeters as the length of the projection of the line connecting symmetric hindlimb distal points (digits 2–4) on the longitudinal axis of the rat. The procedure was repeated six times in immediate succession, and the mean HL-PA value for a given rat was used in statistical analyses. The rat was regarded as asymmetric if the magnitude of HL-PA exceeded the 2-mm threshold (see statistical section). The limb displacing shorter projection was considered as flexed.

In a subset of the rats HL-PA was assessed by the hands-off method. Silk threads were glued to the nails of the middle three toes of both hindlimbs, and their other ends were tied to hooks attached to the movable platform that was operated by a micromanipulator. Positions of the limbs were adjusted to the same, symmetric level, and stretching was performed for a distance of 1–1.5 cm at a rate of 2 cm/s. The threads then were relaxed, the limbs were set free and the resulting HL-PA was photographically recorded. The procedure was repeated six times in succession, and the mean value of postural asymmetry for a given rat was used in statistical analyses. The hands-on and hands-off procedures yielded the same HL-PA magnitude and direction; the MPA correlated with the asymmetry of the force applied to stretch the contralesional and ipsilesional hindlimbs (Watanabe H and Bakalkin G, unpublished observations; Zhang et al., 2020). Experimenters were blinded to the experimental conditions where possible.

### Spinal cord transection and decerebration

The animals were anesthetized with sodium pentobarbital (i.p.; 60 mg/kg body weight, as an initial dose and then 6 mg/kg every hour), or with isoflurane (1.5% isoflurane in a mixture of 65% nitrous oxide and 35% oxygen) anesthesia. After measurement of postural asymmetry, the rats were placed on a stereotaxic frame to maintain the body temperature at 37 ± 0.3°C with a heating pad connected by a rectal probe (CMA150, CMA). A laminectomy at the thoracic T2-T3 level was conducted and the spinal cord was completely transected between the ligatures. Local infiltration of 3.5 mg/ml lidocaine (xylocaine) with 2.2 μg/ml adrenaline was used to reduce nociceptive input during surgery. The completeness of the transection was confirmed by (1) inspecting the cord under microscope during the operation to ensure that no spared fibers bridged the transection site and that the rostral and caudal stumps of the spinal cord are completely retracted; (2) placing a piece of Spongostan (Medispon; MDD) between the rostral and caudal stumps of the spinal cord; and (3) examining the spinal cord after termination of the experiment. After completion of surgical procedures, the wounds were closed with 3–0 suture (AgnTho’s) and rats were kept under infrared radiation lamp to maintain body temperature during monitoring of postural asymmetry.

The decerebration procedure was described elsewhere (Dobson and Harris, 2012). After a craniotomy, the brain rostral to the inferior colliculus was removed with an electrical surgical suction machine (New Askir 20, MedicalExpo). Bleeding was stopped with Meditamp Extra (MDD Dedical Devices). The anesthesia was then discontinued. Experiments were terminated on signs of deterioration, such as a precipitous drop in blood pressure or rise in expiratory CO_2_ level.

### EMG experiments

Four groups consisted of 39 rats including 11 sham and 14 UBI not treated (control) rats, and seven sham and seven UBI rats treated with naloxone rats were analyzed between third and sixth day after UBI or sham surgery. The number of animals which data were used in statistical analysis is shown in Extended Data Figure 3-1.

**Figure 1.**
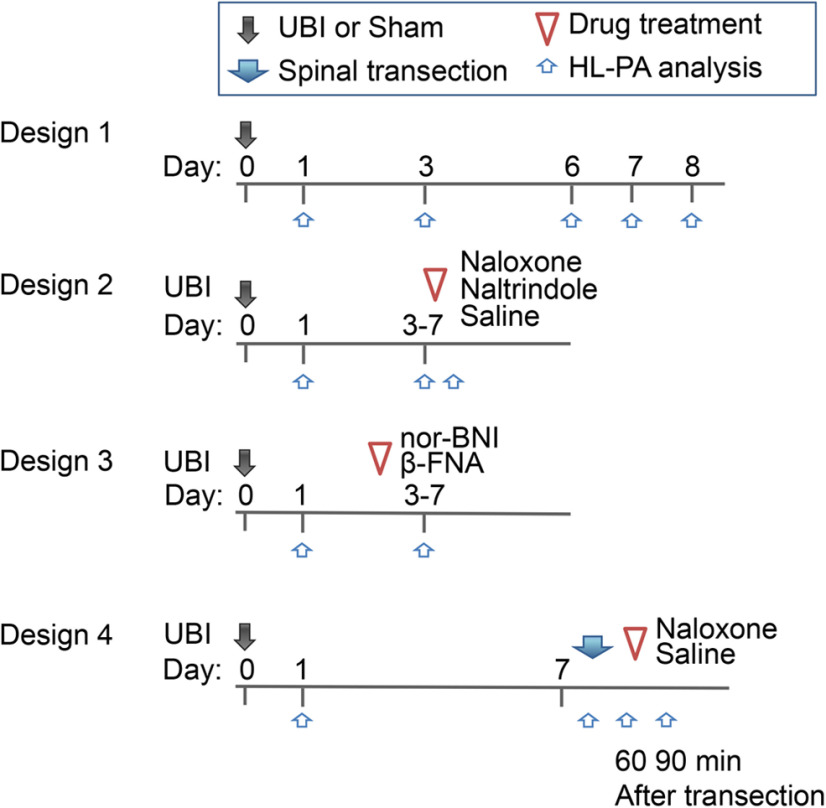
An experimental design. Rats received UBI or sham surgery on day 0. HL-PA was analyzed before and after treatment with opioid antagonists or saline; and before and after complete spinal cord transection. In design 1, a time course of HL-PA after the UBI or sham surgery was analyzed. In design 2, naloxone, NTI, or saline were injected to the left-side and right-side UBI rats. HL-PA was evaluated on day 1, and on the test day before and 30 min after the injection. In design 3, effects of nor-BNI and β-FNA on HL-PA induced by the left-side or right-side UBI were analyzed; the antagonists were administered 1 d before the analysis. In design 4, effects of spinal cord transection, and effects of naloxone in rats with transected spinal cord on the HL-PA were examined.

10.1523/ENEURO.0548-20.2021.f3-1 Extended Data Figure 3-1 The number of rats used in statistical analysis of withdrawal reflexes for each muscle. The rats were exposed to the right-side UBI or the right-side sham surgery, and treated with saline (control group) or naloxone before analysis of reflexes on days 6–8 after the injury. Data for a given muscle were included in statistical models if the number of animals showing EMG response of both the left and right limbs was 5 or more in each of four groups. Altogether 39 rats including 11 sham and 14 UBI not naloxone treated (control) rats, and seven sham and seven UBI naloxone treated rats were analyzed. Download Figure 3-1, DOCX file.

#### Electromyography recordings

Core temperature was maintained between 36 and 38°C using a thermostatically controlled, feedback-regulated heating system. The EMG activity of the extensor digitorum longus (EDL), interossei (Int), and peroneaus longus (PL) muscles of both hindlimbs were recorded. EMG analysis was initiated within ∼3 h after spinalization and decerebration. Recordings were performed using gauge stainless steel disposable monopolar electrodes (DTM-1.00F, The Electrode Store). The electrodes were insulated with Teflon except for ∼200 μm at the tip. The impedance of the electrodes was from 200 to 1000 kΩ. For EMG recordings, a small opening was made in the skin overlying the muscle, and the electrode was inserted into the mid-region of each muscle belly. A reference electrode was inserted subcutaneously in an adjacent skin region. The electrode position was checked by passing trains (100 Hz, 200 ms) of cathodal pulses (amplitude < 30 μA, duration 0.2 ms). The EMG signal was recorded with Spike 2 program (CED) with a sampling rate of 5000 Hz. Low and high pass filter was set at 50,000 and 500 Hz, respectively. Generally, the EMG activity of two or three pairs of hindlimb muscles was recorded simultaneously in each experiment/rat.

#### Cutaneous stimulation

Stimulation sites were decided according to each muscle’s receptive field (Schouenborg et al., 1992; Weng and Schouenborg, 1996). After searching the receptive field of each muscle in responding to pinch stimulation, a pair of stimulation electrodes that were the same as the recording electrodes were inserted subcutaneously into the center of each muscle’s receptive field. The same pairs of digits (i.e., 2, 3, 4, and 5) of both limbs were stimulated to induce ipsilateral reflex responses (Schouenborg et al., 1992). To detect the stimulation intensity that induce the maximal reflex in each muscle, graded current pulses (1 ms, 0.1 Hz) were used ranging mostly from 1–30 mA, occasionally up to 50 mA. The reflex threshold was defined as the lowest stimulation current intensity evoking a response at least in three out of five stimulations. If a muscle response was induced by stimulation at more than one site, the lowest current was taken as a threshold value. For EMG data collection, the current level that induced submaximal EMG responses from both legs, usually at 5–20 mA was chosen. This was usually two to three times higher than the threshold currents. The same current level was used on symmetrical points from the most sensitive area on both paws. For each site EMG responses from 18–20 stimulations at 0.1-Hz frequency were collected. No visible damage of the skin, or marked changes in response properties at the stimulation sites, were detectable at these intensities.

#### EMG data analysis

The spikes from Spike2 EMG data files were sorted with Offline Sorter (version 3, Plexon). The EMG amplitude (spike number) from different muscles was calculated with NeuroExplorer (Nex Technologies). To avoid stimulation artifacts, spikes from the first one or two stimulations were removed from further analysis. The number of spikes was calculated from 16 consecutive stimulations thereafter. The EMG thresholds and responses registered from 0.2 to 1.0 s were analyzed. In this animal preparation, the withdrawal reflexes evoked by innocuous stimulation were very weak compared with those evoked by noxious stimulation (Schouenborg et al., 1992; Weng and Schouenborg, 1998; Schouenborg, 2002; Zhang et al., 2020).

#### Criteria for comparison of the left and right hindlimbs

Strict criteria were applied to ensure data comparability between two hindlimbs for (1) the experimental procedures including the symmetry of stimulation and recording conditions between the two sides; (2) application of electrodes with similar resistance for analysis of symmetric muscles; (3) selection of the reflex characteristics for the analysis; and (4) statistical analysis. The core criteria were similar with those described previously (Hultborn and Malmsten, 1983a,b; Malmsten, 1983). Pairs of each stimulation and recording electrodes were positioned as symmetrically as possible. Stimulation electrodes were inserted into the center of the receptive fields of the left and right muscles (Schouenborg et al., 1992; Weng and Schouenborg, 1996), and recording electrodes into the middle portion of the muscle belly. This was performed by an experienced investigator with knowledge in anatomy and physiology. The same stimulation patterns were used for stimulation of pairs of digits to induce reflexes in symmetric muscles. The same threshold level was used for the left and right muscles. In general, the current level that was applied exceeded by 2- to 3-fold the higher threshold recorded for a muscle in the pair at a given stimulation site. Data recorded with stimulation of more than one site (digits 2, 3, 4, and 5) were processed as replicates to decrease experimental error. Only ipsilateral responses were recorded. Only data for pairs of muscles of the same animal were included in the analysis.

To minimize interindividual variations that may be caused by differences in physiological and experimental conditions, including depth of anesthesia, and circulatory and respiratory states, the asymmetry index calculated for each animal but not absolute values of the reflex size (i.e., reflex amplitude, thresholds and the number of spikes), was analyzed. Comparison between the left and right sides using the asymmetry index was based on the assumption that one side was a reference for the other side in each animal, and this approach largely diminished contributions of the interindividual variations (Hultborn and Malmsten, 1983a,b; Malmsten, 1983).

Analysis of the asymmetry index allowed double assessment, first, within each UBI and control (sham surgery) group that identified the asymmetric versus symmetric pattern for a given group; and, second, between the animal groups that revealed the UBI versus sham surgery differences, and the naloxone versus control differences. Analysis of the asymmetry index in the control group established whether the observed distribution was close to the expected symmetric patterns (that is the assessment of the size of variations around the symmetry point) and, therefore, demonstrated the validity of the approach.

Because multiple responses were measured for the same animal, including two limbs, three muscles, and differing stimulation conditions, and because they were analyzed within animal groups and between the groups treated with and without naloxone, we applied mixed-effects models using Bayesian inference. To avoid bias in the acquisition of experimental observations that may be imposed by intermediate data analyses, the data processing and statistical analysis were performed after completion of all experiments.

### Analysis of gene expression

Intact rats and rats exposed to the left-side or right-side UBI or the left-side or right-side sham surgery (five groups; *n* = 10/group) were killed by decapitation on day 3 after the injury. The lumbar spinal cord was dissected into the left and right halves. The tissue samples were snap frozen and stored at −80°C until assay. In the replication study two independent groups of rats with the left sham-injury (*n* = 11) and left UBI (*n* = 12 rats) were used in analysis of lateralization.

#### Quantitative RT-PCR

Total RNA was purified by using RNeasy Lipid Tissue Mini kit (QIAGEN). RNA concentrations were measured with Nanodrop (Nanodrop Technologies). RNA (500 ng) was reverse-transcribed to cDNA with the cDNA iScript kit (Bio-Rad Laboratories) according to manufacturer’s protocol. cDNA samples were aliquoted and stored at −20°C. The cDNAs were mixed with PrimePCR Probe assay (Extended Data Fig. 5-1) and iTaq Universal Probes supermix (Bio-Rad Laboratories) for qPCR with a CFX384 Touch Real-Time PCR Detection System (Bio-Rad Laboratories) according to the manufacturer’s instructions. The following conditions were applied for the 2-step real-time PCR reaction: 3 min at 95°C, then 40 cycles for 5 s at 95°C, followed by incubation for 30 s at 60°C.

10.1523/ENEURO.0548-20.2021.f5-1 Extended Data Figure 5-1 Genes analyzed and PCR Probes for their analysis (Bio-Rad Laboratories). Download Figure 5-1, DOCX file.

All quantitative RT-PCR procedures were conducted strictly in accordance with the established guidelines for the qRCR based analysis of gene expression, the minimum information for publication of quantitative real-time PCR experiments guidelines (MIQE; Bustin et al., 2009; Taylor et al., 2019). The raw qRT-qPCR data were obtained by the CFX Maestro Software for CFX384 Touch Real-Time PCR Detection System (Bio-Rad Laboratories). The mRNA levels of genes of interest were normalized to the geometric mean of expression levels of two reference genes *Actb* and *Gapdh* selected out of 10 genes (*Actb*, *B2m*, *Gapdh*, *Gusb*, *Hprt*, *Pgk*, *Ppia*, *Rplpo13a*, *Tbp*, and *Tfrc*), using the geNorm program (https://genorm.cmgg.be/; Vandesompele et al., 2002; Kononenko et al., 2017, 2018). The expression stability of candidate reference genes was computed for ten sets of samples that were the left and right halves of the lumbar spinal cord obtained from intact rats, left-sided and right-sided sham surgery groups, and the left-sided and right-sided UBI groups, and was as follows (from high to low): *Actb*, *Gapdh*, *Tbp*, *Rplpo13a*, *Hprt*, *Pgk*, *B2m*, *Tfrc*, *Ppia*, and *Gusb*. In each experiment, the internal reference gene-stability measure M value did not exceed the 1.5 threshold value imposed by the MIQE. The number of reference genes was optimized using the pairwise stability measure (V value) calculated by the geNorm program. The V value for *Actb* and *Gapdh*, the top reference genes did not exceed the 0.15 threshold demonstrating that analysis of these two genes is sufficient for normalization.

A proportion in the expression of subtypes of opioid receptor genes and those in the opioid peptide precursor genes were analyzed as the *Oprd1*/*Oprm1*, *Oprk1*/*Oprd1*, and *Oprk1*/*Oprm1* ratio, and the *Pdyn*/*Penk* ratio, respectively. These ratios did not depend on expression levels of reference gens and normalization procedure.

#### Neuroplasticity-related genes (Extended Data Fig. 5-1)

*Arc*, activity-regulated cytoskeletal gene implicated in numerous plasticity paradigms; *Bdnf*, brain-derived neurotrophic factor regulating synaptogenesis; *cFos*, a neuronal activity dependent transcription factor; *Dlg4* gene codes for PSD95 involved in AMPA receptor-mediated synaptic plasticity and post-NMDA receptor activation events; *Egr1* regulating transcription of growth factors, DNA damage, and ischemia genes; *Gap-43* coding for growth-associated protein Gap-43 that regulates axonal growth and neural network formation; *GluR1* and *Grin2b* coding for the glutamate ionotropic receptor AMPA type Subunit 1 and NMDA receptor subunit, respectively, both involved in glutamate signaling and synaptic plasticity; *Grin2a* subunit of the glutamate receptors that regulates formation of neural circuits and their plasticity; *Homer-1* giving rise to Homer Scaffold Protein 1, a component of glutamate signaling involved in nociceptive plasticity; *Nfkbia* (I-κ-B-α) that inhibits NF-κ-B/REL complexes regulating activity-dependent inhibitory and excitatory neuronal function; *Syt4* (synaptotagmin 4) playing a role in dendrite formation and synaptic growth and plasticity; and *Tgfb1* that gives rise to transforming growth factor β1 regulating inflammation, expression of neuropeptides and glutamate neurotoxicity, were selected as representatives of neuroplasticity-related genes (Anderson and Winterson, 1995; Hayashi et al., 2000; Buisson et al., 2003; Joynes et al., 2004; You et al., 2004; Adkins et al., 2006; O’Mahony et al., 2006; Tappe et al., 2006; Vavrek et al., 2006; Larsson and Broman, 2008; Dolan et al., 2011; Santibañez et al., 2011; Grasselli and Strata, 2013; Harris et al., 2016; Won et al., 2016; Epstein and Finkbeiner, 2018).

### Radioimmunoassay (RIA)

The procedure was described elsewhere (Christensson-Nylander et al., 1985; Merg et al., 2006). Briefly, 1 M hot acetic acid was added to finely powdered frozen tissues, and samples were boiled for 5 min, ultrasonicated and centrifuged. Tissue extract was run through SP-Sephadex ion exchange C-25 column, and peptides were eluted and analyzed by RIA. Cross-reactivity of Leu-enkephalin-Arg (LER) antiserum with dynorphin B and Leu- and Met-enkephalin was <0.1% molar, with α-neoendorphin 0.5% molar, with Dynorphin A (1–8) 0.7% molar, with Met-enkephalin-Arg-Phe (MEAP) 1% molar and with Met-enkephalin-Arg 10% molar. Cross-reactivity of MEAP antiserum with Met-enkephalin, Met-enkephalin-Arg, Met-enkephalin-Arg-Gly-Leu, Leu-enkephalin and LER was <0.1% molar (Nylander et al., 1997). This RIA variant readily detected LER in wild-type mice (Nguyen et al., 2005) but not in *Pdyn* knock-out mice; thus the assay was highly specific and not sensitive to the presence of contaminants. The peptide content is presented in fmol/mg tissue. No normalization per amount of tissue was performed for calculation of the LER/MEAP ratio that therefore was free of any bias because of differences in amount of tissue between the analyzed animal groups, and between the left and right spinal halves.

### Experimental time line/drug treatment design (Fig. 1)

The HL-PA in UBI rats was analyzed before and after antagonist administration. The rats with the MPA >2 mm were defined as asymmetric; the 2-mm MPA was 94th percentile after the sham surgery. Rats that were defined as asymmetric (89% in UBI group) on day 1, were used for the following analysis of the antagonists or saline.

#### Design 1

HL-PA induced by the left or right-side UBI and its time course. The HL-PA was examined on days 1, 3, 6, 7, and 8 after the UBI, and on days 1 and 3 after sham surgery.

#### Design 2

Analysis of effects of naloxone, naltrindole (NTI) and saline. HL-PA formation in rats with the left or right-side UBI was examined on day 1 (control measurement 1), and then before (control measurement 2) and 30 min after administration of naloxone, NTI, or saline on day 3, 5, or 7 after the operation.

#### Design 3

Analysis of effects of nor-binaltorphimine (nor-BNI) and β-funaltrexamine (β-FNA). Antagonists were administered 1 d before HL-PA analysis that was conducted on day 3, 5, or 7 after the left or right-side UBI. Control HL-PA measurement was performed on day 1.

#### Design 4

Comparison of HL-PA in rats before and after transection of the spinal cord. Effects of naloxone on the HL-PA in rats with transected spinal cord. The spinal cord was transected on day 7; HL-PA was measured before and 1 h after the transection and then naloxone or saline were administered and HL-PA was analyzed 30 min later.

Naloxone (10 mg/kg, i.p.), NTI (5 mg/kg, i.p.), and saline were administered 30 min before analysis of HL-PA, while nor-BNI (6 mg/kg, s.c.) and β-FNA (3 mg/kg, s.c.) 1 d before the analysis. In the EMG experiments, naloxone (10 mg/kg) was infused 30 min before the analysis.

Doses and timeline for naloxone (Norris et al., 2009), NTI (Petrillo et al., 2003; Nizhnikov et al., 2009; Rutten et al., 2018), nor-BNI (Horan et al., 1992; Patkar et al., 2013; Rutten et al., 2018) and β-FNA (Petrillo et al., 2003) were robustly established in previous studies to block the respective receptors. The dose for naloxone was chosen to block all three subtypes of opioid receptors. nor-BNI and β-FNA exert long-lasting antagonistic effects that persist for at least one month and are receptor selective from day 1 after administration.

### Statistical analysis

#### HL-PA and electrophysiology

Processing and statistical analysis of the HL-PA, EMG, and molecular data were performed after completion of the experiments by the statisticians, who were not involved in execution of experiments. Therefore, the results of intermediate statistical analyses could not affect acquisition of experimental data.

Postural asymmetry and EMG data were analyzed using Bayesian regression models via full Bayesian framework by calling *Stan* 2.21 (Carpenter et al., 2017) from *R* 3.6.3 R Core Team using the *brms* 2.12 (Burkner, 2017) interface. Predictors and outcomes were centered and scaled. To reduce the influence of outliers, models used Student’s *t* response distribution family with identity link function. Models had no intercepts with indexing approach to predictors (McElreath, 2019). According to Stan recommendations (Gelman, 2019) weakly informative priors were used for group-level effects, residual SD and group-level SD *p* values, adjusted using the multivariate t distribution with the same covariance structure as the estimates, were produced by frequentist summary in *emmeans* 1.4.6 (Searle et al., 1980). Medians of the posterior distribution and 95% highest posterior density continuous intervals (HPDCIs) were plotted. The contrast between groups was defined as significant if both 95% HPDCI did not include zero and adjusted *p* value was ≤0.05.

#### Gene expression and peptides

The mRNA levels of the endogenous opioid system genes (*Pdyn*, *Penk*, *Oprk1*, *Oprd1*, and *Oprm1*) and the levels of opioid peptides (LER, MEAP) in the left and right halves of the lumbar spinal cord were compared among the five rat groups that were control (intact), left side sham, right side sham, left side UBI and right side UBI) rats, respectively. The gene/gene ratios including *Oprk1/Oprd1*, *Oprk1/Oprm1*, *Oprd1/Oprm1*, *Pdyn/Penk* ratios and the LER/MEAP ratio were also compared between the groups. The Kolmogorov–Smirnov and Levene’s tests revealed deviations from normality and differences in the variances between the rat groups, respectively, for several genes and peptides, and therefore the nonparametric Kruskal–Wallis test was used for analysis with group as a factor. The *p* values were corrected for multiple comparisons; a Bonferroni correction factor of 18 and 6 was applied for mRNA and peptides, respectively.

In the absence of significant differences between the five animal groups, as determined by the Kruskal–Wallis test, the groups were combined, and the pooled data were used in analysis of lateralization of the mRNA and peptide levels using Wilcoxon matched pairs signed-rank test. Bonferroni correction factors of nine and three were applied in the RNA and peptide analyses, respectively; adjusted *p* values are shown. Reported fold changes were defined as ratios between median expression levels. In the replication study the sham-injured (*n* = 11) and UBI (*n* = 12 rats) groups did not differ in expression levels, and were combined for analysis of lateralization; unadjusted *p* values are shown.

Differences among animal groups in the gene co-expression patterns that characterize regulatory interactions between the genes (Dobrin et al., 2009; Long et al., 2016; Zhang et al., 2020) were assessed by analysis of the coordination strength as well as the proportion of positive and negative gene-gene correlations (Kononenko et al., 2017; Zhang et al., 2020). Statistical comparison of the coordination strength between the animal groups was performed using Kruskal–Wallis test to sets of squared values of all Spearman’s rank correlation coefficients. Significance of differences in the proportion of positive and negative correlations among animal groups was assessed using the Fisher’s exact test with 2 × 5 and 2 × 2 contingency tables.

In co-expression analysis, intraregional and interregional gene co-expression patterns of the five opioid genes, and those among the five opioid genes and 13 neuroplasticity-related genes (*Arc*, *Bdnf*, *cFos*, *Dlg4*, *Egr1*, *Homer-1*, *Gap43*, *GluR1*, *Grin2a*, *Grin2b*, *Nfkbia*, *Syt4*, and *Tgfb1*) were analyzed. Spearman’s rank correlation coefficient was calculated for (1) pairs of the opioid genes for the left and right spinal halves separately (*n* = 10/each half) and between them (*n* = 25); and (2) pairs of the opioid genes with neuroplasticity-related genes for the left and right spinal halves separately (*n* = 65/each half) and between them (*n* = 130). Sample size for each correlation coefficient was *n* = 10 animals. The significance level was set to *p* ≤ 0.05.

### Data availability

Data supporting the findings of this study are available within the article, its Extended Data, or on request.

### Materials

Naloxone, β-FNA, NTI, and nor-BNI were purchased from Tocris. All test compounds were dissolved in saline in experiments with HL-PA. Naloxone was dissolved in an infusion buffer in the EMG study.

## Results

To examine whether the UBI effects were mediated through the opioid system, pharmacological approach with the general and selective opioid antagonists was used. The UBI was performed by suction lesion to restrict a damaged area to the hindlimb sensorimotor cortex and examine specific changes in hindlimb motor functions. The HL-PA with contralesional flexion and asymmetry of the NWRs were studied as the readouts of the UBI because they induced or regulated differentially on the left or right body side by opioid peptides; and may be directed along the left-right axis, that is essential for analysis of a side-specific regulation.

### Cortical injury sites

The lesion produced by the UBI was confined to the hindlimb motor area (Zhang et al., 2020). The lesion sites extended 3.2–5.0 mm rostrocaudally and 1.8–2.8 mm mediolaterally. The lesion was 1.0–1.5 mm in depth and did not affect the white matter below the cortex. Because of tissue necrosis around the cavity border, the actual lesion size in some cases was slightly larger than the intended dimensions of 3.5 × 2 mm. The average lesion volume was 6.05 ± 1.53 mm^3^ (mean value ± SD), without accounting for tissue shrinkage because of fixation. The lesion volumes of the cortices from the left (5.86 ± 1.86 mm^3^, *n* = 10) and right (6.36 ± 1.22 mm^3^, *n* = 11) UBI rats were similar (*p* = 0.47; two tailed *t* test).

### The UBI-induced HL-PA: effects of the general opioid antagonist naloxone

**Figure 2.**
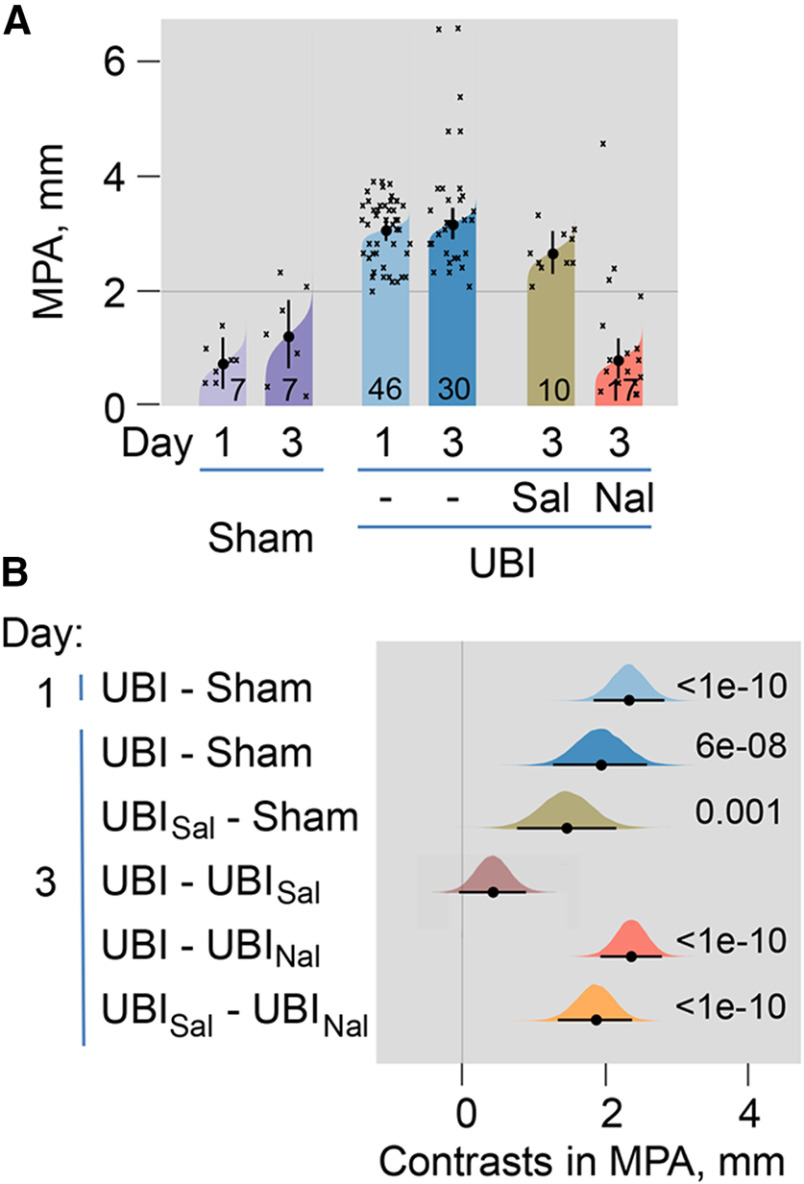
The HL-PA induced by UBI. Effects of the general opioid antagonist naloxone. HL-PA was analyzed in the UBI or sham surgery rats on days 1 and 3; and on day 3 in UBI rats 30 min after treatment of with naloxone or saline. The number of rats is shown on the plots (design 2; naloxone: 14 left and 3 right UBI rats; saline: 5 left and 5 right UBI rats). HL-PA was measured in millimeters as the length of the projection of the line connecting symmetric hindlimb distal points on the longitudinal axis of the rat and presented as the MPA. ***A***, The MPA is plotted as median, 95% HPDCI, and posterior distribution from Bayesian regression. ***B***, Contrasts in MPA between rat groups are plotted as medians, 95% HPDCI and densities from Bayesian sampler. Adjusted *p* values are shown if they are <0.05 for differences in MPA between rat groups. Differences were considered to be significant if 95% HPDCI did not include zero, and adjusted *p* values were <0.05. Time course, comparison of isoflurane and pentobarbital anesthesia, and effects of spinal cord transection and administration of saline or naloxone on HL-PA after the left and right-side UBI are shown on Extended Data Figure 2-1.

The UBI induced HL-PA that was manifested as flexion of the contralesional hindlimb (Figs. 1, 2; Extended Data Fig. 2-1). The MPA was substantially (∼3-fold) greater after UBI compared with sham surgery (Fig. 2). The MPA of the left and right-side UBI groups did not differ at all-time points analyzed and did not change from day 1 to day 8 after the injury in both groups (Extended Data Fig. 2-1*A*). The MPA was virtually the same under isoflurane and pentobarbital anesthesia (Extended Data Fig. 2-1*D*,*E*). After complete spinal cord transection, HL-PA persisted essentially with the same MPA (Extended Data Fig. 2-1*F*,*G*), suggesting that neuroplasticity processes induced by UBI in the lumbar neural circuits underlie the HL-PA maintenance.

10.1523/ENEURO.0548-20.2021.f2-1 Extended Data Figure 2-1 Formation of HL-PA after the left and right-side UBI. Time course, comparison of isoflurane and pentobarbital anesthesia, and effects of spinal cord transection and administration of saline or naloxone. HL-PA was measured in millimeters as the length of the projection of the line connecting symmetric hindlimb distal points (digits 2–4) on the longitudinal axis of the rat and presented as its magnitude (MPA). ***A***, Time course for the left and right UBI-induced HL-PA. HL-PA was analyzed on days 1, 3, 6, 7, and 8 after the UBI. ***B***, ***C***, Saline was injected to the UBI rats on day 3, or day 7 after spinal cord transection 30 min before the HL-PA analysis. ***D***, ***E***, Comparison of the HL-PA in the same rats analyzed under isoflurane and pentobarbital anesthesia on days 1 and 7 after the UBI, respectively. Contrast in MPA between the analyses under isoflurane and pentobarbital anesthesia are shown. ***F***, ***G***, Comparison of the HL-PA in the same rats analyzed before and 1 h after spinal cord transection on day 7 after the UBI. Contrast in MPA between the analyses are shown. ***H–J***, Effects of naloxone (2 left-side and 3 right-side UBI rats) versus saline on HL-PA in rats with transected spinal cord. Naloxone or saline was administered 1 h after transection that was performed on day 7 after the UBI. Contrasts are shown (***I***) for the same rats analyzed before and after naloxone/saline injection, respectively; and (***J***) between the naloxone and saline treated groups analyzed before and after injection. The MPA is plotted as the median and 95% highest density central interval (whiskers = 95% HDCI) in ***A***, ***B***, ***D***, ***F***. The MPA (***H***) and contrasts in MPA between rat groups (***C***, ***E***, ***G***, ***I***, ***J***) are plotted as medians, 95% HPDCI and densities from Bayesian sampler. The number of rats is shown on the plots. Adjusted *p* values are shown if they are <0.05 for differences in MPA between rat groups. Differences were considered to be significant if 95% HPDCI did not include zero, and adjusted *p* values were <0.05. A day of analysis within the 8-d observation period and UBI side showed no effects on MPA in statistical models. No significant differences were evident at all comparisons in ***B–G***; 95% HPDCI did include zero. Download Figure 2-1, TIF file.

To examine whether opioid receptors mediate the UBI-induced formation of HL-PA, the effects of the general opioid antagonist naloxone were analyzed (Fig. 2; Extended Data Fig. 2-1*H–J*). Naloxone or saline was administered to the UBI rats 30 min before the HL-PA analysis (Fig. 1, design 2) in rats with intact (Fig. 2) and transected (Extended Data Fig. 2-1*H–J*) spinal cords. In both experiments naloxone injection resulted in substantial (∼3-fold) decreases in the MPA, suggesting a role of the opioid system in asymmetric spinal processes underlying HL-PA development.

### Effect of naloxone on the hindlimb NWRs in the UBI rats

**Figure 3.**
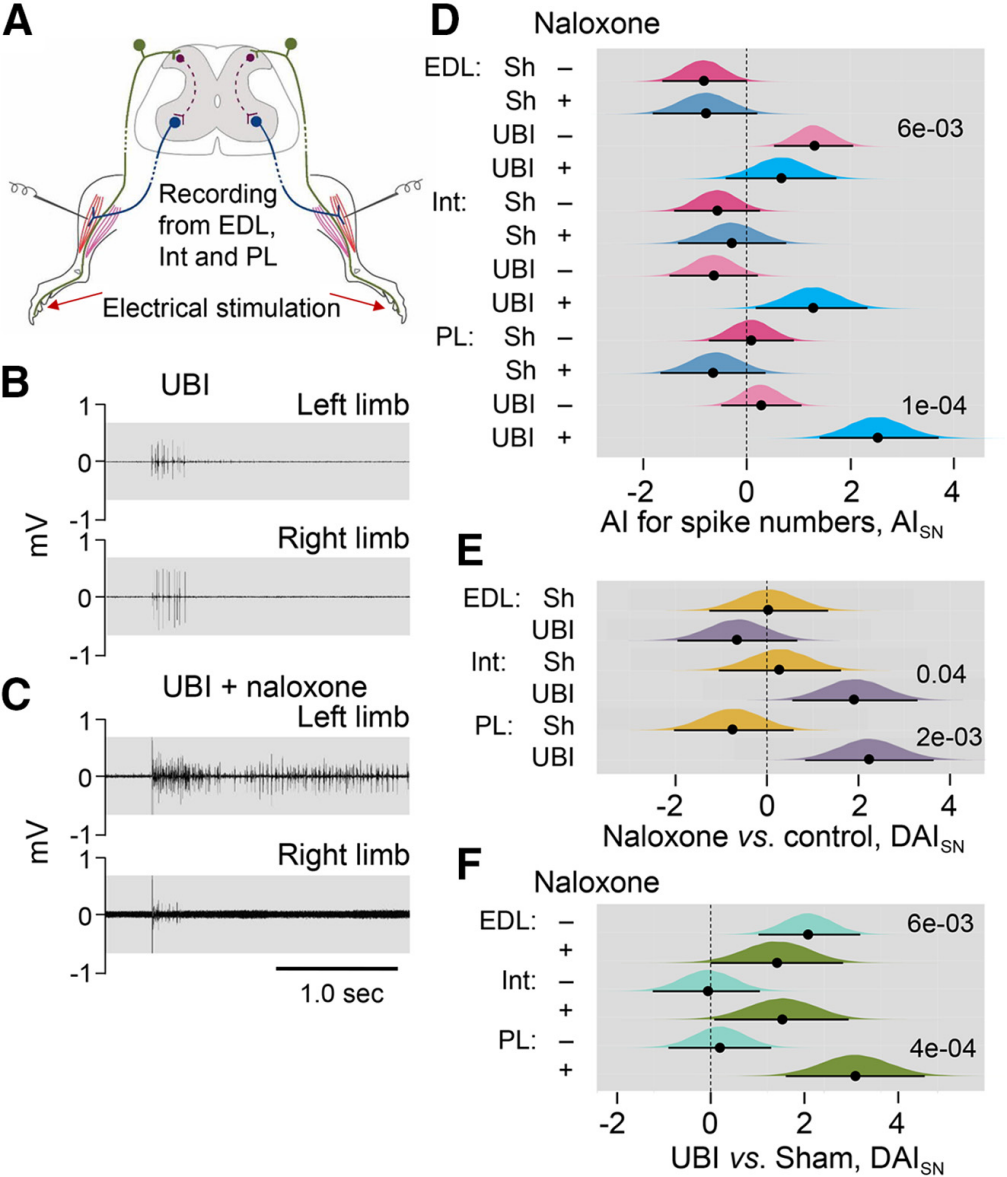
Effects of naloxone on the left-right asymmetry index for the spike number (AI_SN_) of the EDL, Int, and PL muscles in the UBI rats. ***A***, Experimental design. Rats with the right UBI or right sham surgery (Sh) were exposed to complete spinal cord transection and decerebration, and were infused with naloxone or infusion buffer. The NWRs of the left and right limbs were stimulated and recorded ipsilaterally. EMG activity of left and right hindlimb muscles was evoked by electrical stimulation of symmetrical digits of left and right hindlimbs. Four groups consisted of 39 rats including 11 sham and 14 UBI not treated (control) rats, and seven sham and seven UBI rats treated with naloxone rats were analyzed between third and sixth day after UBI or sham surgery. The number of animals which data were used in statistical analysis is shown in Extended Data Figure 3-1. ***B***, ***C***, Representative EMG responses of the Int muscle to electrical stimulation of digit 4 of the UBI rats. ***D***, The left-right asymmetry index for the spike number (AI_SN_ = log_2_[(1 + SN_Left_)/(1 + SN_Right_)]; where Left and Right were values for the left and right side). ***E***, Difference between the naloxone-treated and naloxone-untreated (control) groups in AI_SN_ [ΔAI = (AI_Naloxone_ – AI_Control_)]. ***F***, Difference between the UBI and respective sham surgery groups in AI_SN_ [ΔAI = (AI_UBI_ – AI_sham_)]. Medians, 95% HPDC intervals and densities of posterior estimates from Bayesian sampler are plotted. Asymmetry and contrast between the groups were defined as significant if 95% HPDC intervals for AI_SN_ did not include zero, and adjusted *p* values were <0.05.

Along with changes in hindlimb posture, the UBI produced asymmetry in the hindlimb NWRs (Zhang et al., 2020). To reveal a role of the opioid system, we examined whether naloxone interferes with the hindlimb NWRs evoked by electrical stimulation of symmetric digits of left and right hindlimbs, and recorded ipsilaterally using EMG techniques in unanesthetized decerebrate UBI rats with transected spinal cord (Fig. 3). The EMG responses of the EDL, Int, and PL muscles were analyzed in four groups of rats that had the right-side UBI or right-side sham surgery, and that were or were not treated with naloxone (Fig. 3; Extended Data Fig. 3-1). Effects of the right side UBI were analyzed because they were substantially greater compared with those induced by the left side lesion (Zhang et al., 2020).

Strict criteria were applied to ensure data comparability between two hindlimbs for (1) the experimental procedures including the symmetry of stimulation and recording conditions and positioning of electrodes; (2) the reflex characteristics by analysis of the asymmetry index; and (3) statistical analysis (described in detail in Materials and Methods). The core criteria were similar with those implemented by Hultborn and Malmsten (1983a,b). To minimize interindividual variations, the asymmetry index calculated for each animal but not the absolute values of the reflex size was analyzed. This analysis allowed double assessment that identified the asymmetric versus symmetric patterns, first, within each of the UBI and control groups, and, second, between the animal groups. Because multiple responses were measured for the same animal, including two of its limbs, three muscles, and the varying stimulation conditions, and because they were analyzed within an animal group and between the groups, we applied mixed-effects models using Bayesian inference.

Asymmetry in EMG responses to current stimulation was compared between the rat groups using the left/right asymmetry index (AI = log_2_[L/R], were L and R were values for the left and right limb muscles) for the threshold (AI_Thr_) or the spike number (AI_SN_). When reflexes on the both sides are equal (i.e., the left/right ratio = 1), the asymmetry index is zero; if reflexes are of double size on the Left or Right side (i.e., the left/right ratio = 2.0 or 0.5) the asymmetry index is +1 or −1, respectively.

The number of analyzed animals is shown in Extended Data Figure 3-1. There were no differences from zero in the AI_Thr_ for the UBI and sham surgery groups, infused with either naloxone or with control solution. Moreover, there were no differences in AI_Thr_ among these groups. No asymmetries in the number of evoked spikes (AI_SN_) were revealed in the sham surgery rats that were treated with naloxone or control solution, with the exception of EDL that showed a higher number of spikes on the right versus left side in the group infused with control solution with borderline significance (median of the posterior distribution was −0.83, 95% HPDCI = [−1.63, −0.01]; Fig. 3*D*). In the UBI group, significant asymmetry in the number of spikes in rats infused with control solution was identified for the EDL muscle (median = 1.30, 95% HPDCI = [0.53, 2.06], *p* =0.006, the left/right ratio = 2.5; Fig. 3*D*), and in naloxone-treated rats for the PL (median = 2.52, 95% HPDCI = [1.41, 3.71], *p* = 1e-04, the left/right ratio = 5.7). The Int demonstrated asymmetry in the number of spikes with borderline significance (median = 1.28, 95% HPDCI = [0.18, 2.33], the left/right ratio = 2.4) in UBI rats.

No effects of naloxone in comparison with respective control (contrast: naloxone vs control) were evident in rats with sham surgery whereas blockade of opioid receptors in the UBI rats resulted in increase in the spike number on the left (contralesional) compared with the right (ipsilesional) side for the Int (median = 1.92, 95% HPDCI = [0.56, 3.29], *p* = 0.04, left/right ratio = 3.8) and PL (median = 2.24, 95% HPDCI = [0.83, 3.64], *p* = 0.01, difference in the left/right ratio = 4.7; Fig. 3*E*). Representative examples of the UBI-induced asymmetry for the Int muscle are shown on Figure 3*B*,*C*. The UBI and sham surgery groups treated with naloxone (contrast: UBI versus sham surgery) were different for the PL (median = 3.18, 95% HPDCI = [1.66, 4.72], *p* = 4e-04, difference in the left/right ratio = 9.1) and for the Int with borderline significance (median = 1.57, 95% HPDCI = [0.08, 3.04], difference in the left/right ratio = 3.0; Fig. 3*F*). The contrast between the UBI and sham surgery in the group received control solution, was significant for the EDL (median = 2.14, 95% HPDCI = [1.05, 3.30], *p* =0.002, difference in the left/right ratio = 4.4).

In summary, naloxone did not produce noticeable effects on (1) the left-right balance in stimulation thresholds of the three muscles in each UBI and sham surgery groups, and (2) differences in this balance between these groups. No asymmetry in the spike numbers in sham surgery groups treated with naloxone or control solution was evident. However, naloxone administration to UBI rats produced asymmetric patterns in the NWRs of the PL and Int characterized by a higher activity on the contralesional (left) compared with ipsilesional (right) side. Thus, the UBI effects may be counteracted by the endogenous opioid peptides resulting in maintenance of the symmetric patterns of the PL and Int NWRs. Naloxone by blocking these effects, unmasked the cryptic lateralized changes induced by the UBI.

### UBI effects on expression of the opioid genes and their co-expression patterns with neuroplasticity genes in the lumbar spinal cord

Formation of HL-PA and asymmetry in the NWRs after the UBI may require neuroplastic rearrangements based on changes in expression of opioid and/or neuroplasticity genes in the lumbar spinal cord. It has been demonstrated that the UBI modified the expression of neuroplasticity genes, as well as robustly impaired coordination of their expression within and between the ipsilesional and contralesional halves of the lumbar spinal cord (Zhang et al., 2020). Here, we assessed whether left and right-side UBI affects expression of the five opioid genes [the μ-receptor, δ-receptor, and κ-receptor genes, *Oprm1*, *Oprd1*, and *Oprk1*, respectively, and the prodynorphin (*Pdyn*) and proenkephalin (*Penk*) opioid peptide precursor genes], and their co-expression patters with 13 neuroplasticity genes (*Arc*, *Bdnf*, *cFos*, *Dlg4*, *Egr1*, *Homer-1*, *Gap43*, *GluR1*, *Grin2a*, *Grin2b*, *Nfkbia*, *Syt4*, and *Tgfb1*) in the lumbar spinal cord. We also examined whether expression of the opioid genes is lateralized in the lumbar spinal cord assuming that the asymmetric expression may be a basis for the left and right side specific opioid effects on HL-PA formation after the UBI. The left and right spinal cord may differ in the level of opioid receptor subtypes, in the proportions of these subtypes, in the proportion of the precursor peptide genes giving rise to the endogenous ligands for μ-receptor, δ-receptor, and κ-receptor. To address these issues, the expression levels and the *Oprd1*/*Oprm1*, *Oprk1*/*Oprd1*, and *Oprk1*/*Oprm1* ratio along with the *Pdyn*/*Penk* ratio were compared between the left and right spinal halves. We also analyzed the levels of LER and MEAP, Pdyn and Penk markers, respectively, as well as their ratio. Five groups including the left-side and right-side UBI rats, the left and right-side sham surgery rats and intact rats were analyzed.

**Figure 4.**
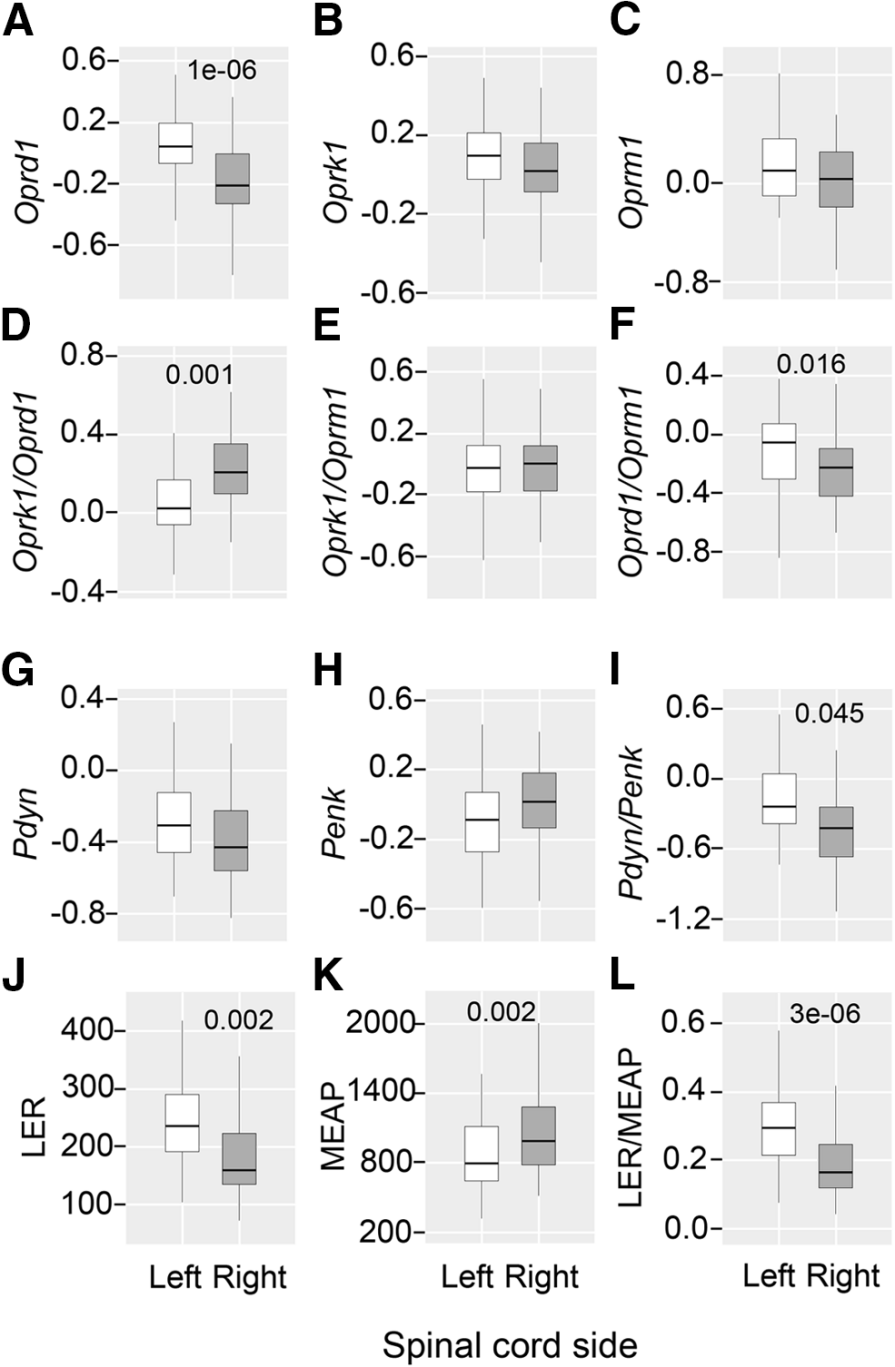
Lateralized expression of the opioid genes and opioid peptides in the lumbar spinal cord. Data for five animal groups were combined separately for each the left and right lumbar spinal halves (*n* = 50), Log_2_-scaled and compared using Wilcoxon matched pairs test. Data are shown for the expression levels of the μ-opioid (*Oprm1*), δ-opioid (*Oprd1*), and κ-opioid (*Oprk1*) receptor genes; the prodynorphin (*Pdyn*) and proenkephalin (*Penk*) opioid peptide precursor genes; and for the *Oprk1*/*Oprd1*, *Oprk1*/*Oprm1*, *Oprd1*/*Oprm1*, and *Pdyn*/*Penk* ratio, along with data for opioid peptides LER and MEAP, the Pdyn and Penk markers, respectively, and for the LER/MEAP ratio. White and dark gray boxes denote the left and right spinal cord. The horizontal line in the box represents the median; the box hinges represent the first (Q1) and third (Q3) quartiles. Upper and lower whiskers extend from the hinge to the highest/lowest value that lies within the 1.5 interquartile range (IQR) of the hinge. The Bonferroni adjusted *p* values are shown. Analysis of replication sample is shown on Extended Data Figure 4-1.

The Kruskal–Wallis test did not reveal differences among the five animal groups in the levels of opioid mRNAs and of the two opioid peptides, in mRNA ratio for opioid receptors and opioid peptide precursors, as well as for the LER/MEAP ratio. Next, to assess whether the opioid system is lateralized in the lumbar spinal cord, the data for the five groups were combined into the left and right datasets (*n* = 50 rats) and compared using the Wilcoxon matched pairs test. Analysis revealed significantly higher expression levels of the *Oprd1* receptor gene (1.19-fold between the medians; *p* = 1.2e-06), as well as higher *Oprd1*/*Oprm1* (1.12-fold; *p* = 0.016) and *Pdyn*/*Penk* (1.14-fold; *p* = 0.045) ratios in the left compared with the right spinal cord (Fig. 4*A*,*F*,*I*). In contrast, the *Oprk1*/*Oprd1* ratio (1.14-fold; *p* = 0.001) was higher on the right spinal side (Fig. 4*D*).

10.1523/ENEURO.0548-20.2021.f4-1 Extended Data Figure 4-1 Analysis of replication sample. Lateralized expression of the opioid genes in the lumbar spinal cord. Data for two animal groups (the left side sham and UBI rats; no differences between the groups in the opioid gene expression were revealed) were combined separately for each the left and right lumbar spinal halves (*n* = 23), Log2-scaled and compared using Wilcoxon matched pairs test. White and dark grey boxes denote the left and right spinal cords. The horizontal line in the box represents the median; the box hinges represent the first (Q1) and third (Q3) quartiles. Upper and lower whiskers extend from the hinge to the highest/lowest value that lies within the 1.5 interquartile range (IQR) of the hinge. Unadjusted *p* values are shown. Download Figure 4-1, TIF file.

The same results were obtained in the replication study (*n* = 23). The analyzed group consisted of the sham-injured (*n* = 11) and UBI (*n* = 12) rats taken from another experiment. These subgroups did not differ in expression levels and were pooled for analysis of lateralization. The Wilcoxon matched pairs test showed significantly higher expression of the *Oprd1* gene (1.39-fold between the medians; *p* = 3.0e-04), and the *Oprd1*/*Oprm1* (1.24-fold; *p* = 0.001) and *Pdyn*/*Penk* (1.24-fold; *p* = 0.019) mRNA ratio in the left compared with the right spinal half (Extended Data Fig. 4-1*A*,*B*,*D*). In opposite, the proportion of the *Oprk1* to *Oprd1* mRNA was significantly higher on the right versus left side (Extended Data Fig. 4-1*C*; 1.12-fold; *p* = 0.028).

Analysis of opioid peptides by the Wilcoxon matched pairs test revealed significantly higher levels of LER (1.48-fold; *p* = 0.002), and the LER/MEAP ratio (1.77-fold; *p* = 4.4e-06) in the left compared with the right spinal half (Fig. 4*J*,*L*). The MEAP levels were significantly higher (1.24-fold; *p* = 0.002) in the right spinal half compared with the left spinal half (Fig. 4*K*).

Gene co-expression patterns characterize regulatory interactions within and across tissues (or CNS areas; Dobrin et al., 2009; Long et al., 2016; Zhang et al., 2020). Here, we assessed whether the left and right UBI affect co-expression patterns of the opioid genes in the left and right halves of the lumbar spinal cord, and between them. Furthermore, we analyzed whether the UBI affects the co-expression patterns of the opioid genes with the neuroplasticity-related genes. We assumed that the opioid system that modulates activity of neurocircuits and neuroplasticity processes may be co-regulated. Spearman’s rank correlations among the expression levels of the five opioid genes; and among the expression levels of the five opioid genes and 13 neuroplasticity-related genes were calculated. The gene-gene coordination strength and the proportion of positive correlations were compared among the naive group, left and right sham groups, and left and right UBI groups.

**Figure 5.**
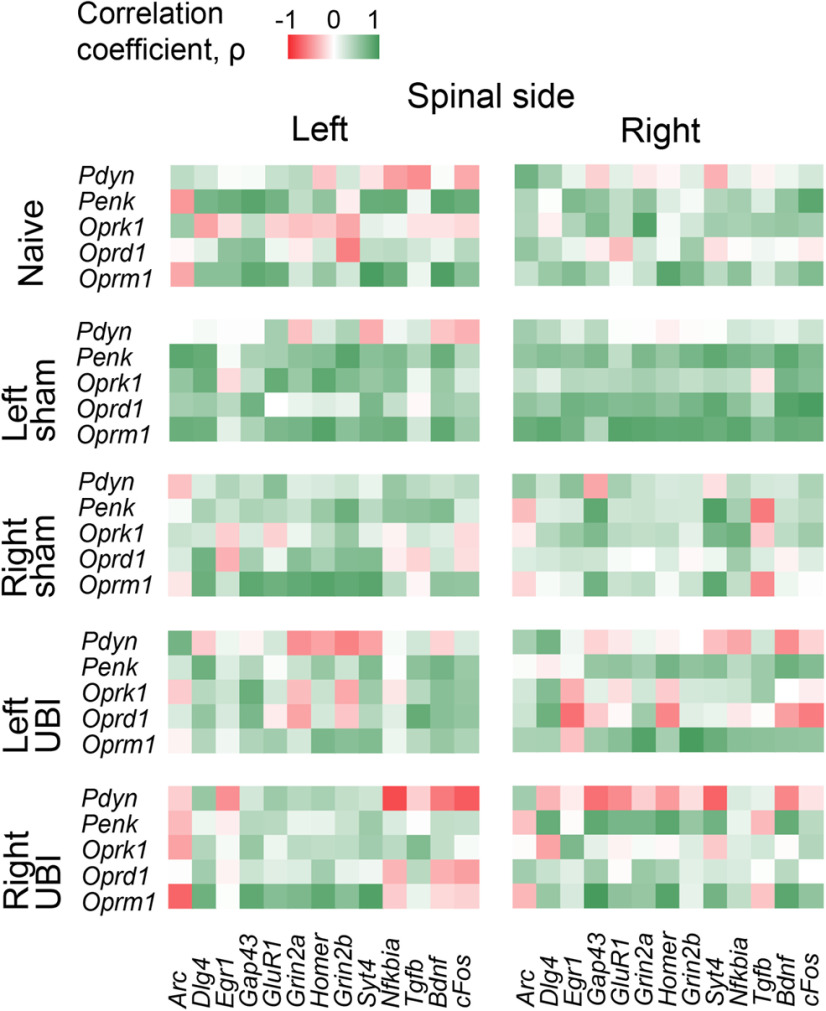
Effects of UBI on intra-area correlations between the opioid and neuroplasticity-related genes (genes analyzed are shown in Extended Data Fig. 5-1) in the left and right halves of the spinal cord. Heatmap for intra-area Spearman’s rank correlations between expression levels of five opioid system genes and 13 neuroplasticity-related genes in the left and right spinal cord for the naive group, the left and right sham surgery groups, and left and right UBI groups (five groups; *n* = 10 rats/group). Significance of differences in the proportion of positive and negative correlations among five groups, and, separately between two animal groups, were assessed by the Fisher’s exact test with 2 × 5 or 2 × 2 contingency tables, respectively. Two-tailed uncorrected *p* value is shown. Differences among five animal groups for the left and right spinal halves: *p* = 0.048 and *p* = 8.4e-05, respectively. Differences for the right spinal half between the left UBI versus left sham surgery: *p* = 2.4e-04; and between the right UBI versus right sham surgery: *p* = 0.043.

Generally, no significant differences in the coordination strength (in other words, in the aggregated level of co-regulation) were observed between animal groups both for the opioid genes and the opioid-vs-neuroplasticity genes. Comparison of the coordination strength did not take into account a sign of correlations, and therefore a separate analysis was performed to assess differences in the proportion of positive and negative correlations between the animal groups. No differences in the proportion of positive intra-area and inter-area correlations for the opioid genes were found between the groups. In contrast, the number of positive intra-area correlations of opioid genes with neuroplasticity-related genes for the right half of the spinal cord was significantly different (*p*_unadjusted_ = 8.4e-05) between the five animal groups while for the left half was nominally significant (*p*_unadjusted_ = 0.048; Fig. 5). In the right spinal cord, the proportion of positive correlations was lower in the left UBI rats versus left sham surgery group (*p*_unadjusted_ = 2.4e-04), and in the right UBI rats versus right sham surgery group with nominal significance (*p*_unadjusted_ = 0.043).

For individual genes, differences between UBI groups and sham groups were substantial for *Pdyn*; UBI significantly reduced the proportion of *Pdyn* positive correlations correlations. The effect was more pronounced for the right-side injury (*p*_unadjusted_ = 3.9e-04; in the analysis correlations were combined for the left and right halves of the spinal cord). Effects of the left-side impact was at the trend level.

No group effect was evident for the proportion of positive correlations at the inter-area comparison. In summary, UBI does not affect the expression of the opioid genes but may induce the opioid system-mediated changes in expression of neuroplasticity-related genes.

### Effect of selective opioid antagonists on formation of HL-PA induced by the left and right-side UBI

**Figure 6.**
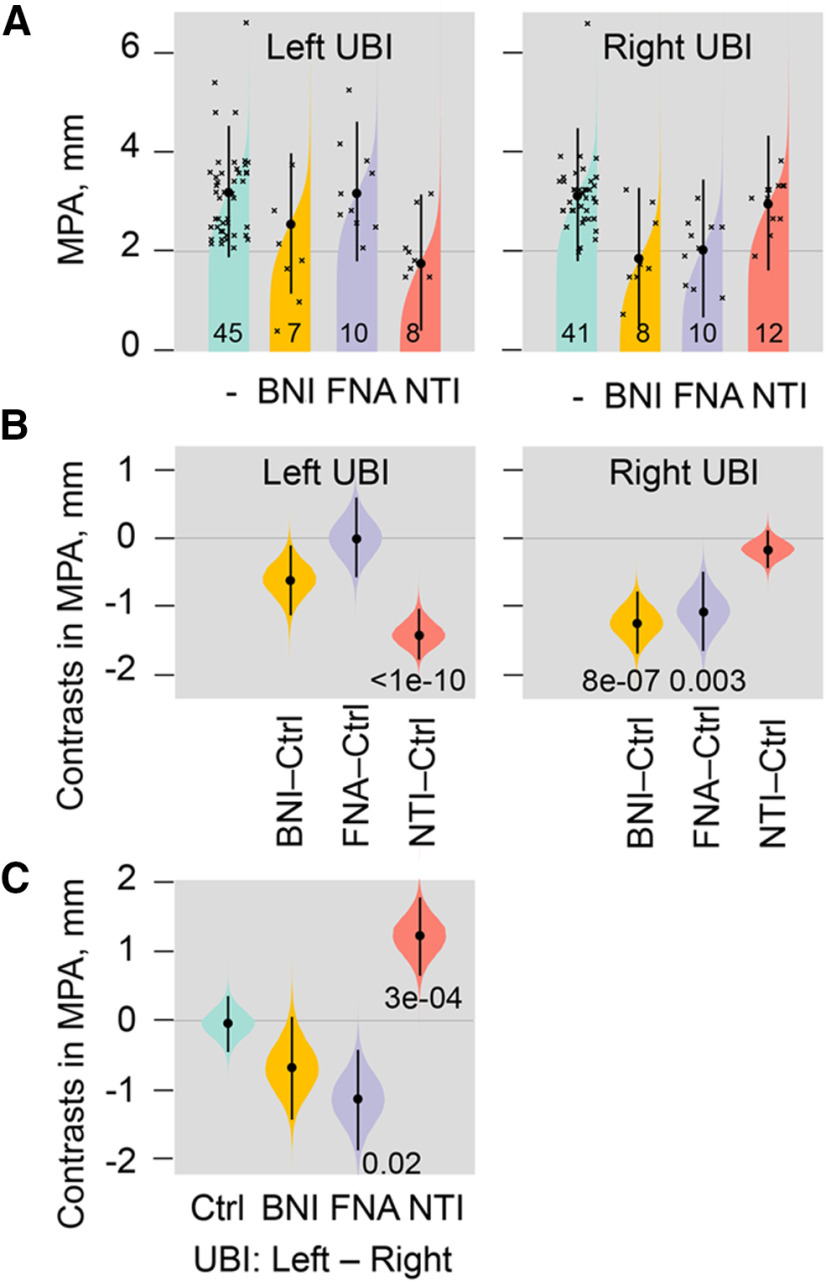
Effects of nor-BNI, β-FNA, and NTI, the selective κ-opioid, μ-opioid, and δ-opioid antagonists, respectively, on HL-PA induced by the left and right UBI. The UBI rats were treated with nor-BNI (BNI) or β-FNA (FNA) 1 d before (design 3), or with NTI 30 min before (design 2) HL-PA analysis. ***A***, The MPA is plotted as median, 95% HPDCI, and posterior distribution from Bayesian regression. The number of rats is shown on the plot. ***B***, Contrasts in MPA between a rat group treated with a given antagonist and respective saline group (Ctrl). ***C***, Contrasts in MPA between rat groups with the left or right-side UBI; the UBI rats in each pair of groups were treated with the same antagonist or saline (Ctrl). Contrasts are plotted as medians, 95% HPDCI and densities from Bayesian sampler. Adjusted *p* values are shown if they are <0.05 for differences in MPA between the groups. Differences were considered to be significant if 95% HPDCI did not include zero, and adjusted *p* values were <0.05.

In order to identify subtypes of the opioid receptors mediating the UBI effects we analyzed HL-PA after administration of μ-antagonist β-FNA, δ-antagonist NTI, and κ-antagonist nor-BNI (Fig. 6). Effects were compared between rats with the left and right-side UBI. nor-BNI and β-FNA are long acting antagonists that selectively block these receptor subtypes, but require ∼24 h after administration to do so (Horan et al., 1992; Petrillo et al., 2003; Patkar et al., 2013; Rutten et al., 2018). These antagonists were administered to the UBI rats 24 h before HL-PA analysis (Fig. 1, design 3). NTI was injected 30 min before the HL-PA analysis (Fig. 1, design 2).

In the left-side UBI group, no substantial effects of β-FNA and nor-BNI were evident while a marked (∼2-fold) decrease in the MPA was induced by NTI (Fig. 6*A*,*B*). In contrast, administration of β-FNA and nor-BNI, but not NTI, resulted in substantial MPA reduction (∼2-fold) in the right-side UBI group (Fig. 6*A*,*B*). Strikingly, effects of NTI and β-FNA were different between the left and right side UBI groups (Fig. 6*C*). Thus, β-FNA and nor-BNI inhibited formation of the left hindlimb flexion, whereas NTI, in contrast, inhibited formation of the right hindlimb flexion suggesting that effects of the right UBI are mediated through μ-receptors and κ-receptors, while those of the left UBI by δ-receptors.

## Discussion

The findings of this study were that the general opioid antagonist naloxone and the selective antagonists of opioid receptors blocked the UBI-induced formation of HL-PA. Furthermore, naloxone administration revealed the cryptic contra-ipsilesional asymmetry in NWR induced by the UBI in the PL and Int muscles. We also found that the opioid gene expression and opioid peptides were lateralized in the lumbar spinal cord, and that coordination of the opioid and neuroplasticity-related gene expression was dysregulated after the UBI.

**Figure 7.**
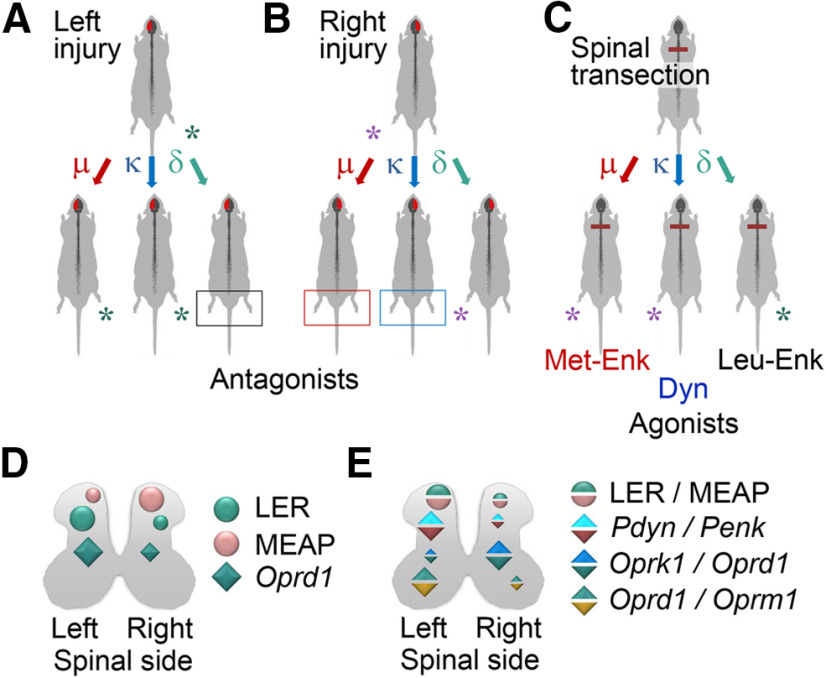
The left and right side-specific effects of opioid antagonists and agonists and the underlying lateralization of the opioid system. ***A***, ***B***, Effects of selective μ-opioid (β-FNA), κ-opioid (nor-BNI), and δ-opioid (NTI) antagonists on HL-PA induced by the left or right side UBI. ***C***, Development of HL-PA with the left or right hindlimb flexion induced by the preferential endogenous μ-agonist Met-enkephalin (Met-Enk), κ-agonist dynorphin (Dyn), and δ-agonist Leu-enkephalin (Leu-Enk; data from Bakalkin et al., 1980; Chazov et al., 1981; Bakalkin and Kobylyansky, 1989). ***D***, Lateralization of opioid peptides LER, the Pdyn marker, and MEAP, the Penk marker, and the lateralized expression of the δ-opioid receptor in the lumbar spinal cord. ***E***, Differences in ratio of (1) LER and MEAP; (2) mRNA levels of their precursor proteins (*Pdyn*/*Penk*), respectively; (3) mRNA levels of κ-opioid and δ-opioid receptors (*Oprk1*/*Oprd1*); and (4) mRNA levels of δ-opioid and μ-opioid receptors (*Oprd1*/*Oprm1*). The findings suggest that the mirror symmetric neural circuits mediating effects of the left and right brain injury on the contralesional hindlimb responses are differentially regulated by the lateralized opioid system.

The selective opioid antagonists had differing effects, depending on which side of the brain had the UBI, that was the striking finding (Fig. 7*A*,*B*). The μ-antagonist β-FNA and κ-antagonist nor-BNI, reduced the magnitude of the asymmetry after the right but not left UBI. In contrast, the δ-antagonist NTI inhibited HL-PA induced by the left-side but not right-side brain injury. These findings are consistent with the observations that opioid peptides and synthetic opioids may induce HL-PA in rats and cats with intact brain, and that the flexion side is determined by the agonist administered (Fig. 7*C*; Bakalkin et al., 1980; Chazov et al., 1981; Bakalkin and Kobylyansky, 1989; Pilyavskii et al., 2013; Watanabe et al., 2020). The left hindlimb was flexed after administration of the μ-/δ-agonist Met-enkephalin, and the selective κ-agonists dynorphin and U-50488 (Bakalkin et al., 1980; Chazov et al., 1981; Bakalkin and Kobylyansky, 1989; Watanabe et al., 2020). Conversely, Leu-enkephalin, which acts via the δ-receptor, caused the right hindlimb flexion (Bakalkin et al., 1980; Chazov et al., 1981). Together, the antagonist and agonist data suggest that the right side UBI-induced formation of the left flexion is mediated by the μ-receptor and κ-receptor targeted by endogenous Met-enkephalin and dynorphins, respectively. Complementarily, development of HL-PA with right flexion induced by the left UBI may be mediated by δ-receptor targeted by Leu-enkephalin (Fig. 7).

These findings corroborate the recent observations that selective opioid antagonists modulate the effects of the controlled cortical impact, a model of clinical focal TBI (Watanabe et al., 2020). Similar to the present study, administration of naloxone and β-FNA but not NTI blocked HL-PA induced by the unilateral right-sided TBI. However, the κ-antagonists nor-BNI and LY2444296 did not affect the magnitude of the asymmetry after the right side TBI, while the side of flexion occurred was reversed. Instead of contralesional (left) hindlimb flexion the ipsilesional (right) limb was flexed (Watanabe et al., 2020). Thus, κ-antagonists differently interfered with the effects of the right-side TBI and the right-side UBI. The UBI and the TBI also differed in the sidedness of their effects on hindlimb posture (Bakalkin et al., 2021). The effects of the left and right UBI were mirror symmetric. In contrast, both the left and right side TBI induced flexion of the left limb. The differences between the injury models may be because of differences in their pathophysiological mechanisms. While UBI effects are more local and confined to the hindlimb sensorimotor area, the TBI in addition to the cortical areas also damages the white matter and underlying structures (Hall et al., 2005, 2008; Ng and Lee, 2019; Lacalle-Aurioles et al., 2020). The TBI effects are caused by mechanical stress-induced axonal and vascular injuries followed by neuronal degeneration and excitotoxicity. These effects are mediated by regulatory molecules that may be similarly activated by the left and right TBI. Some of these molecules may be involved in lateralized processes and produce side specific effects because of lateralization of their target receptors. Thus, the opioid peptides dynorphins are affected by both the left or right side injury (Hauser et al., 2005; Hussain et al., 2012; Kononenko et al., 2018), and may induce a side-specific response, i.e., flexion of the left hindlimb (Bakalkin and Kobylyansky, 1989; Watanabe et al., 2020). Inhibition of this response by κ-antagonists could reverse the flexion side if other neurohormones that induce a response on the other (right) side are also activated by the TBI (Watanabe et al., 2020). In contrast to this mechanism, the more local UBI may primarily affect the left or right sided neuronal circuits of the mirror-symmetric neural pathways that convey signals from the injured hemisphere to lumbar motoneurons. These pathways may be locally controlled at different levels of the neuraxis by the lateralized opioid system, and their targeting by selective opioid antagonists may lead to activation or inactivation of their left or right-sided lumbar neurocircuits.

The HL-PA and NWRs were used as the UBI readouts because the opioid system has a role in their formation and regulation, and because they are directed along the mediolateral axis allowing analysis of left-right sided processes. The HL-PA with contralesional flexion is a proxy for hindlimb responses to a unilateral brain lesion and is developed both in animals under anesthesia and un-anesthetized decerebrate animals (Watanabe et al., 2020; Zhang et al., 2020). Previous studies established that the stretch and postural limb reflexes were substantially inhibited by anesthesia (Zhou et al., 1998; Fuchigami et al., 2011) and abolished by spinal cord transection (Miller et al., 1996; Musienko et al., 2010; Frigon et al., 2011). In HL-PA studies, no nociceptive stimulation was applied and tactile stimulation was negligible (DiGiorgio, 1929; Chamberlain et al., 1963; Zhang et al., 2020). Thus, the nociceptive withdrawal and stretch reflexes likely did not contribute to HL-PA formation in the UBI rats that were examined under anesthesia both before and after spinal transection.

The polysynaptic NWRs may undergo pathologic changes after brain injury because of the aberrant activity or injury of descending pathways that converge with inputs from peripheral afferents (Crenna and Frigo, 1984; Duysens et al., 1990, 1992; Rossi and Decchi, 1994; Andersen et al., 1995; Spaich et al., 2004, 2006; Sandrini et al., 2005; Emborg et al., 2009). Asymmetrically exacerbated withdrawal reflexes develop often and lead to flexor spasms in patients after stroke and TBI (Dewald et al., 1999; Serrao et al., 2012; Spaich et al., 2014). The NWR-related EMG kinematic responses were increased and NWR modulation impaired in patients with hemiparesis (Serrao et al., 2012; Alvisi et al., 2018). UBI produced asymmetric patterns of the NWR of the EDL muscle with higher activity on the contralesional versus ipsilesional side (Zhang et al., 2020), while the Int and PL reflexes were still symmetric after the injury. However, blockade of opioid receptors by naloxone revealed a marked asymmetry of the NWR of these two muscles with higher (∼4-fold) responses on the contralesional versus ipsilesional side. Thus, the UBI effects were cryptic; the opioid system likely counteracted the UBI-induced maladaptive changes through inhibition of the contralesional and/or activation of ipsilesional NWRs that resulted in maintenance of the symmetric NWR patterns. In animals with sham surgery, the opioid system probably was not involved in coordination of the contra-ipsilesional balance of the PL, Int, and EDL NWRs.

Naloxone effects were evident in the UBI animals with transected spinal cord suggesting that asymmetric hindlimb posture and reflexes are controlled by spinal neural circuits regulated by the endogenous opioid peptides. In the spinal cord, the μ-opioid, δ-opioid, and κ-opioid receptors are expressed both in the dorsal and ventral horns (Kononenko et al., 2017; Wang et al., 2018). The δ-opioid receptor is expressed in multiple classes of neurons that regulate spinal motor control while the δ-receptors and μ-receptors are co-expressed in V1 ventral horn interneurons (Wang et al., 2018). Opioid agonists exert their action on ventral root reflexes via presynaptic inhibition of afferent signaling, postsynaptic inhibition of the dorsal horn interneurons and actions on ventral horn interneurons regulating motoneurons activity (Wang et al., 2018). This may result in suppression of the ipsilateral reflexes (Faber et al., 1997) while targeting of opioid receptors in neurons surrounding the central canal (Mansour et al., 1994; Wang et al., 2018) may inhibit the spinal commissural pathways (Light and Perl, 1979; Petkó et al., 2004) and contralateral reflexes (Duarte et al., 2019). Opioid peptides suppressed reflexes evoked by electrical stimulation of the skin (Clarke et al., 1992; Steffens and Schomburg, 2011) that may attenuate pain and to promote healing (Steffens and Schomburg, 2011). Spinal circuits that mediate the asymmetric UBI effects may be controlled by the same opioid mechanisms.

The side-specific opioid effects suggest that the opioid receptor subtypes are lateralized in the spinal cord, and that the asymmetrically distributed receptors differentially regulate the mirror-symmetric spinal neural circuits that control the left and right hindlimb muscles. Asymmetric expression of the opioid genes was earlier reported for the cervical spinal cord (Kononenko et al., 2017). All three opioid receptors were lateralized to the left while their proportions were different between the left and right spinal halves. The expression profiles were coordinated between the dorsal and ventral domains but differently on the left and right sides. The present study identified generally the same lateralization patterns in the lumbar spinal cord (Fig. 7*D*,*E*). Expression of the δ-receptors was lateralized to the left whereas a proportion of κ-receptors and δ-receptors (the *Oprk1*/*Oprd1* expression ratio) was higher on the right side. Opioid peptides were also lateralized. The levels of LER (a prodynorphin marker), and the LER to MEAP (a proenkephalin marker) ratio, and, consistently, the ratio of prodynorphin to proenkephalin mRNA were greater in the left half compared with the right half. MEAP was lateralized to the right. Lateralization of the opioid system in the spinal cord may provide a molecular basis for differential regulation of the UBI-induced left and right sided processes.

The previous study demonstrated that the UBI robustly impaired coordination of expression of neuroplasticity-related genes within and between the ipsilesional and contralesional halves of the lumbar spinal cord (Zhang et al., 2020). Among these genes, expression of the *Grin2a*, *Dlg4*, and *Tgfb1* genes associated with the glutamate system was affected. The *Grin2a* subunit of the glutamate receptors regulates formation and plasticity of neural circuits. The *Dlg4* gene gives rise to PSD95 involved in post-NMDA receptor activation events (Won et al., 2016). *Tgfb1* codes for transforming growth factor β1 regulating glutamate neurotoxicity (Buisson et al., 2003; Santibañez et al., 2011). The present study did not reveal UBI-induced changes in the expression of the opioid genes. However, their co-expression pattern with neuroplasticity-related genes was dysregulated as evident from the decrease in the proportion of positive gene-gene correlations after the UBI. The decrease was robust in the right half of the spinal cord especially after the left UBI. Changes in coordination of the opioid–neuroplasticity-related gene expression is a novel phenomenon suggesting that the UBI effects on neuroplastic processes are mediated by the opioid system.

The side-specific antagonist effects on HL-PA were revealed in anaesthetized animals that limited the understanding of biological and clinical role of this phenomenon. Electrophysiological mechanisms of the HL-PA formation and clinical correlates of this asymmetry have not been yet addressed. Nonetheless, the HL-PA exhibits several features of the human upper motor neuron syndrome induced by TBI or stroke. First, they include the asymmetric pattern with deficits on the contralesional side. Second, formation of contralesional flexion correlates with the asymmetric hindlimb motor impairments in locomotor tasks (Lukoyanov N, Bakalkin G, unpublished observations). Third, HL-PA may depend on the efferent drive but not on the afferent stimulation because it is resistant to bilateral lumbar deafferentation (Zhang et al., 2020). In this feature, HL-PA is similar with “spastic dystonia,” a tonic muscle overactivity that contributes to “hemiplegic posture” in patients (Gracies, 2005; Sheean and McGuire, 2009; Lorentzen et al., 2018). Another limitation is that the pathways from the injured brain to the spinal motor circuits that are targeted by opioid antagonists have not been identified. These clinical and mechanistic issues are beyond the scope of this report on the bipartite neurohormonal regulation, and should be addressed in further studies.

Functional specialization of the left and right hemispheres is an organizing principle of the brain (MacNeilage et al., 2009; Concha et al., 2012; Duboc et al., 2015; Güntürkün et al., 2020; Vallortigara and Rogers, 2020). The computational advantages of the specialization may include paralleled processing of information modules in the left and right hemispheres that increases the flow of information, and improves functional performance. Lasting regulation of the lateralized processes may be accomplished by local paracrine molecules, including neuropeptides, that may preferentially operate either on the left or right side of the midline (Zink et al., 2011; Hussain et al., 2012; Marlin et al., 2015; Watanabe et al., 2015, 2020; Kononenko et al., 2017, 2018; Nation et al., 2018; Phelps et al., 2019). Our findings suggest a more general role for the lateralized neuropeptide systems than regulation of the lateralized functions. We hypothesize that the left-side and right-side specific neurohormonal mechanism regulate the non-lateralized, mirror-symmetric neural circuits that control paired organs and extremities including the left and right hindlimbs. Neuropeptides may differentially target the left and right counterparts of these circuits and, in this way, control left-right balance in their functional performance. This bipartite mechanism may be based on lateralization of the neurohormonal systems, and may operate locally (e.g., within the lumbar spinal cord), or at the several levels of the neuraxis by controlling neural pathways that convey signals from the left and right hemispheres to the contralateral hindlimb motoneurons. A unilateral brain lesion may shift this balance to the left or to the right, depending on the side of injury, that impairs the left-right side specific neurohormonal control leading to asymmetric functional deficits.

In conclusion, this study presents evidence for the opioid neurohormonal mechanism that may mediate effects of a UBI on spinal motor circuits and determine whether the left or right hindlimb is affected. This mechanism was revealed by analysis of selective opioid antagonists which effects were found to be specific for the injury side. These findings corroborate the observations that the endogenous opioid peptides and selective synthetic agonists of opioid receptors may mimic the effects of unilateral brain lesion by producing the left and right hindlimb postural responses in animals with intact brains (Bakalkin et al., 1980, 1986; Chazov et al., 1981; Bakalkin and Kobylyansky, 1989; Watanabe et al., 2020). This mechanism may rely on the lateralized opioid receptors and opioid peptides in the lumbar spinal cord, and on coordination of expression of the opioid and neuroplasticity-related genes. Together these findings suggest that mirror-symmetric neural circuits that mediate the effects of left and right brain injury on the contralesional hindlimbs are differentially controlled by the lateralized opioid system.

Several experimental and clinical studies demonstrated that the general opioid antagonists naloxone and naltrexone may decrease spasticity caused by progressive multiple sclerosis (Gironi et al., 2008) and lessen neurologic deficits developed after cerebral ischemia (Baskin and Hosobuchi, 1981; Hosobuchi et al., 1982; Baskin et al., 1984, 1994; Jabaily and Davis, 1984; Namba et al., 1986; Skarphedinsson et al., 1989; Hans et al., 1992; Wang et al., 2019). It is important to identify clinical and pathophysiological features of sensorimotor impairments and postural deficits that are mediated by the opioid receptor subtypes, and to establish whether pharmacological targeting of these features may contribute to sensorimotor recovery and compensate for postural deficits after TBI and stroke. From a biological standpoint, it is interesting to ascertain whether the neurohormonal mechanisms may serve to control a balance between the left–right processes in bilaterally symmetric animals.

## Synthesis

Reviewing Editor: Michael Michaelides, NIDA-NIH Decisions are customarily a result of the Reviewing Editor and the peer reviewers coming together and discussing their recommendations until a consensus is reached. When revisions are invited, a fact-based synthesis statement explaining their decision and outlining what is needed to prepare a revision will be listed below. The following reviewer(s) agreed to reveal their identity: Renata Marchette, Jeffery Boychuk.

Reviewer 1

The manuscript Left-right side-specific neuropeptide mechanism mediates contralateral responses to a unilateral brain injury presents novel and interesting findings on the lateralized nociceptive and somatosensorial deficits following unilateral brain injury. The authors present a robust study with very interesting results, in which the care dedicated to experimental design is very clear.

But there are some points that need to be addressed for the manuscript to be ready for publication.

Introduction:

The authors should consider reordering and expanding the introduction section and start by contextualizing the importance of their study in the field before discussing the role of the opioid system in the somatosensorial asymmetric responses.

Methods:

Considering it is an extensive study it would be easier to follow if the methods were described by experiment from the start.

- At what time do the lights turn on in the vivarium?

- In which phase of the cycle are the experimental procedures carried out?

- The naloxone (10 mg/kg) dose used is high, was this intentional to act on all opioid receptors?

- Avoid using terms such as ‘sacrifice’, euthanasia is the appropriate term to be used.

Discussion:

The first paragraph of the discussion should summarize the findings instead of providing the study’s rationale and the discussion should be focused on tying the findings back to the literature

Conclusion:

Should be the conclusion of the findings in the present study.

Reviewer 2

The following is a review for the manuscript entitled: “Left-right side-specific neuropeptide mechanism mediates contralateral responses to a unilateral brain injury.” This study investigates how unilateral brain injury affects spinal circuitry/signaling and has implicated opioid signaling in this process. It is a careful study with clear reporting of approach and results. This work is timely and highly relevant to field-specific research and the broader neuroscience community. Recommendations are listed below.

1. Please include additional previous studies on this topic (and further distinguish this new study from this literature). A non-exhaustive list of similar studies is provided here. In particular, doi.org/10.1093/braincomms/fcaa208 is similar but appears to differ in injury model, opioid pharmacology and outcome measures.

Ipsilesional versus contralesional postural deficits induced by unilateral brain trauma: a side reversal by opioid mechanism

https://academic.oup.com/braincomms/article/2/2/fcaa208/6007254

https://doi.org/10.1093/braincomms/fcaa208

Opioids induce postural asymmetry in spinal rat: the side of the flexed limb depends upon the type of opioid agonist

https://pubmed.ncbi.nlm.nih.gov/2540878/

doi: 10.1016/0006-8993(89)90193-5

Dynorphin B induces lateral asymmetric changes in feline spinal cord reflexes

https://www.frontiersin.org/articles/10.3389/fnins.2013.00244/full

https://doi.org/10.3389/fnins.2013.00244

## Author Response

Dear Drs. Bernard and Michaelides,

We thank the reviewers and the editor for their well-considered and encouraging analysis of our

manuscript. We agree with the comments and suggestions, and have addressed them to the best

of our ability. We responded to each of the points raised by the reviewers below.

General Comments

"In an effort to improve rigor, transparency, and reproducibility, eNeuro strongly encourages the

use of estimation statistics.”

RESPONSE: We applied estimation statistics for analysis of the results when it was necessary

(please, see Figures 2, 3 and 6, and Extended Figure 2-1), and would appreciate if this could be

highlighted to increase the exposure of our paper.

"Comments on the Visual Abstract for Author (Required): The visual abstract can be improved by

providing a legend to define the colored boxes and to highlight that hindlimb postural asymmetry

is a key outcome since the images of this asymmetry may be missed by readers if they are brief in

viewing the article. The visual abstract does not present any of the gene expression findings. Figure

5 is very interesting so ideally this information is somehow conveyed in the visual abstract.”

RESPONSE: Thank you for the suggestion. We have prepared a new Visual Abstract and included

it in the resubmission files.

Reviewer #1:

1. “Introduction: The authors should consider reordering and expanding the introduction section

and start by contextualizing the importance of their study in the field before discussing the role of

the opioid system in the somatosensorial asymmetric responses.

"

RESPONSE: We thank the reviewer for this remark. The introduction has been rewritten

accordingly, and now it provides background information with the importance of the study,

hypothesis, and aims. At the same time, we could not further expand it for more comprehensive

justification of the study in the context of the lateralization and TBI fields, due to the limit in 750

words imposed by the journal.

2. “Methods: Considering it is an extensive study it would be easier to follow if the methods were

described by experiment from the start. - At what time do the lights turn on in the vivarium? - In

which phase of the cycle are the experimental procedures carried out? - The naloxone (10 mg/kg)

dose used is high, was this intentional to act on all opioid receptors? - Avoid using terms such as

‘sacrifice’, euthanasia is the appropriate term to be used.”

RESPONSE: All these points have been addressed throughout the manuscript.

2 (2)

3. “Discussion: The first paragraph of the discussion should summarize the findings instead of

providing the study’s rationale and the discussion should be focused on tying the findings back to

the literature"

RESPONSE: The discussion has been substantially modified accordingly to the remark. The

findings and their novelty and originality are now discussed in the first place, and in the contexts of

early and very recent publications on the topic.

4. “Conclusion: Should be the conclusion of the findings in the present study.”

RESPONSE: The conclusion has been rewritten, and now it covers the finding, concept,

and their clinical and biological relevance.

Reviewer #2:

1. “Please include additional previous studies on this topic (and further distinguish this new study

from this literature). A non-exhaustive list of similar studies is provided here. In particular,

doi.org/10.1093/braincomms/fcaa208 is similar but appears to differ in injury model, opioid

pharmacology and outcome measures. (Ipsilesional versus contralesional postural deficits induced

by unilateral brain trauma: a side reversal by opioid mechanism:

https://academic.oup.com/braincomms/article/2/2/fcaa208/6007254;

https://doi.org/10.1093/braincomms/fcaa208; Opioids induce postural asymmetry in spinal rat:

the side of the flexed limb depends upon the type of opioid agonist,

https://pubmed.ncbi.nlm.nih.gov/2540878/ doi: 10.1016/0006-8993(89)90193-; Dynorphin B

induces lateral asymmetric changes in feline spinal cord reflexes,

https://www.frontiersin.org/articles/10.3389/fnins.2013.00244/full,

https://doi.org/10.3389/fnins.2013.00244"

RESPONSE: We thank the reviewer for the list of references and the suggestion. In the

resubmitted manuscript, the findings are discussed in the context of the aforementioned studies,

and novelty, differences and similarity of the results along with mechanistic causes of the

differences among the studies, are emphasized.

Thank you for your consideration,

Sincerely.
