# Supplementary material for: Left-Right Side-Specific Neuropeptide Mechanism Mediates Contralateral Responses to a Unilateral Brain Injury
Source: eNeuro. 2021 May 22;8(3):ENEURO.0548-20.2021. doi: 10.1523/ENEURO.0548-20.2021 (PMC8152370; doi:10.1523/ENEURO.0548-20.2021)
Supplement: Extended Data Figure 3-1 — The number of rats used in statistical analysis of withdrawal reflexes for each muscle. The rats were exposed to the right-side UBI or the right-side sham surgery, and treated with saline (control group) or naloxone before analysis of reflexes on days 6–8 after the injury. Data for a given muscle were included in statistical models if the number of animals showing EMG response of both the left and right limbs was 5 or more in each of four groups. Altogether 39 rats including 11 sham and 14 UBI not naloxone treated (control) rats, and seven sham and seven UBI naloxone treated rats were analyzed. Download Figure 3-1, DOCX file. [file enu-eN-NWR-0548-20-s03.docx]

**Figure 3-1. The number of rats used in statistical analysis of withdrawal reflexes for each muscle.**

| **Muscle** | **Sham rats** | | **UBI rats** | |
| --- | --- | --- | --- | --- |
|  | **Control** | **Naloxone** | **Control** | **Naloxone** |
| EDL | 9 | 6 | 10 | 6 |
| Int | 8 | 5 | 5 | 6 |
| PL | 9 | 7 | 9 | 6 |

The rats were exposed to the right-side UBI or the right-side sham surgery, and treated with saline (control group) or naloxone before analysis of reflexes on Day 6, 7 or 8 after the injury. Data for a given muscle were included in statistical models if the number of animals showing EMG response of both the left and right limbs was 5 or more in each of four groups. Altogether 39 rats including 11 sham and 14 UBI not naloxone treated (control) rats, and 7 sham and 7 UBI naloxone treated rats were analyzed.
