# Supplementary material for: Left-Right Side-Specific Neuropeptide Mechanism Mediates Contralateral Responses to a Unilateral Brain Injury
Source: eNeuro. 2021 May 22;8(3):ENEURO.0548-20.2021. doi: 10.1523/ENEURO.0548-20.2021 (PMC8152370; doi:10.1523/ENEURO.0548-20.2021)
Supplement: Extended Data Figure 5-1 — Genes analyzed and PCR Probes for their analysis (Bio-Rad Laboratories). Download Figure 5-1, DOCX file. [file enu-eN-NWR-0548-20-s05.docx]

**Figure 5-1. Genes analyzed and PCR Probes for their analysis (Bio-Rad Laboratories, CA, USA).**

| Gene | Gene symbol | Assay ID | Channel |
| --- | --- | --- | --- |
| *Actin Beta* | *Actb* | qRnoCIP0050804 | HEX |
| *Activity-regulated cytoskeleton-associated protein* | *Arc* | qRnoCEP0027389 | HEX |
| *Arginine vasopressin receptor 1A* | *Avpr1a* | qRnoCEP0023750 | FAM |
| *Brain-derived neurotrophic factor* | *Bdnf* | qRnoCEP0026843 | HEX |
| *Fos proto-oncogene* | *cFos* | qRnoCEP0024078 | HEX |
| *Discs large MAGUK scaffold protein 4* | *Dlg4* | qRnoCIP0026242 | FAM |
| *Early growth response 1* | *Egr1* | qRnoCEP0022872 | FAM |
| *Growth associated protein 43* | *Gap43* | qRnoCIP0027599 | FAM |
| *Glyceraldehyde-3-phosphate dehydrogenase* | *Gapgh* | qRnoCIP0050838 | HEX |
| *Glutamate ionotropic receptor AMPA type subunit 1* | *GluR1* | qRnoCIP0030725 | FAM |
| *Glutamate ionotropic receptor NMDA type subunit 2a* | *Grin2a* | qRnoCIP0025244 | HEX |
| *Glutamate ionotropic receptor NMDA type subunit 2b* | *Grin2b* | qRnoCIP0023973 | HEX |
| *Homer scaffold protein 1* | *Homer-1* | qRnoCEP0023985 | FAM |
| *Opioid receptor delta 1* | *Oprd1* | qRnoCEP0029668 | FAM |
| *Opioid receptor kappa 1* | *Oprk1* | qRnoCIP0029310 | HEX |
| *Opioid receptor mu 1* | *Oprm1* | qRnoCEP0024902 | FAM |
| *Prodynorphin* | *Pdyn* | qRnoCEP0025357 | FAM |
| *Proenkephalin* | *Penk* | qRnoCEP0029455 | HEX |
| *Proprotein convertase subtilisin/kexin type 6* | *Pcsk6* | qRnoCIP0045340 | FAM |
| *NFKB inhibitor alpha* | *Nfkbia* | qRnoCEP0026759 | HEX |
| *Synaptotagmin 4* | *Syt4* | qRnoCIP0029728 | FAM |
| *Transforming growth factor beta 1* | *Tgfb1* | qRnoCIP0031022 | HEX |
