# Supplementary figures and images for: Left-Right Side-Specific Neuropeptide Mechanism Mediates Contralateral Responses to a Unilateral Brain Injury
Source: eNeuro. 2021 May 22;8(3):ENEURO.0548-20.2021. doi: 10.1523/ENEURO.0548-20.2021 (PMC8152370; doi:10.1523/ENEURO.0548-20.2021)

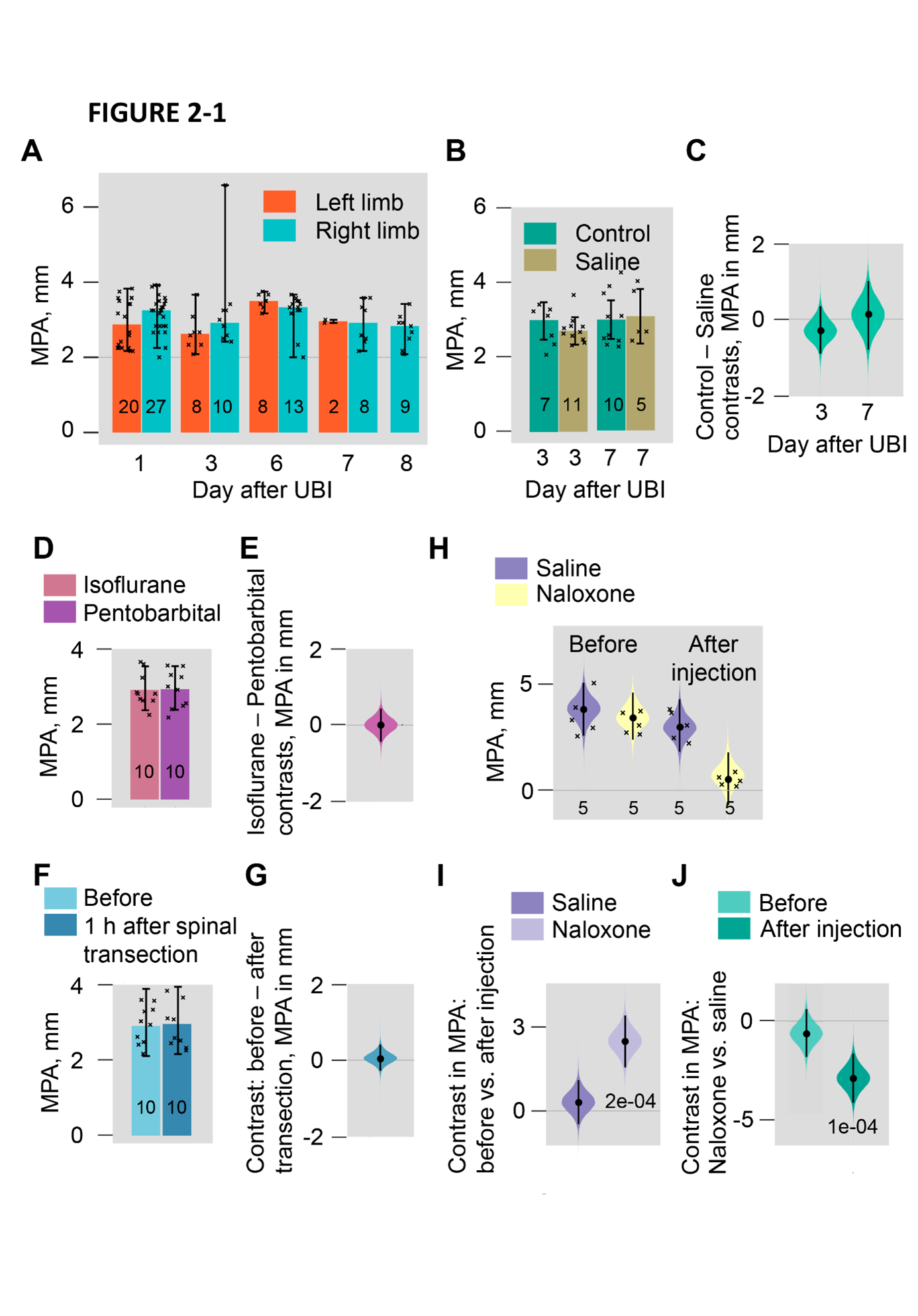

Supplement: Extended Data Figure 2-1 — Formation of HL-PA after the left and right-side UBI. Time course, comparison of isoflurane and pentobarbital anesthesia, and effects of spinal cord transection and administration of saline or naloxone. HL-PA was measured in millimeters as the length of the projection of the line connecting symmetric hindlimb distal points (digits 2–4) on the longitudinal axis of the rat and presented as its magnitude (MPA). A, Time course for the left and right UBI-induced HL-PA. HL-PA was analyzed on days 1, 3, 6, 7, and 8 after the UBI. B, C, Saline was injected to the UBI rats on day 3, or day 7 after spinal cord transection 30 min before the HL-PA analysis. D, E, Comparison of the HL-PA in the same rats analyzed under isoflurane and pentobarbital anesthesia on days 1 and 7 after the UBI, respectively. Contrast in MPA between the analyses under isoflurane and pentobarbital anesthesia are shown. F, G, Comparison of the HL-PA in the same rats analyzed before and 1 h after spinal cord transection on day 7 after the UBI. Contrast in MPA between the analyses are shown. H–J, Effects of naloxone (2 left-side and 3 right-side UBI rats) versus saline on HL-PA in rats with transected spinal cord. Naloxone or saline was administered 1 h after transection that was performed on day 7 after the UBI. Contrasts are shown (I) for the same rats analyzed before and after naloxone/saline injection, respectively; and (J) between the naloxone and saline treated groups analyzed before and after injection. The MPA is plotted as the median and 95% highest density central interval (whiskers = 95% HDCI) in A, B, D, F. The MPA (H) and contrasts in MPA between rat groups (C, E, G, I, J) are plotted as medians, 95% HPDCI and densities from Bayesian sampler. The number of rats is shown on the plots. Adjusted p values are shown if they are <0.05 for differences in MPA between rat groups. Differences were considered to be significant if 95% HPDCI did not include zero, and adjusted p values were <0.05. A day of [file enu-eN-NWR-0548-20-s02.tif]

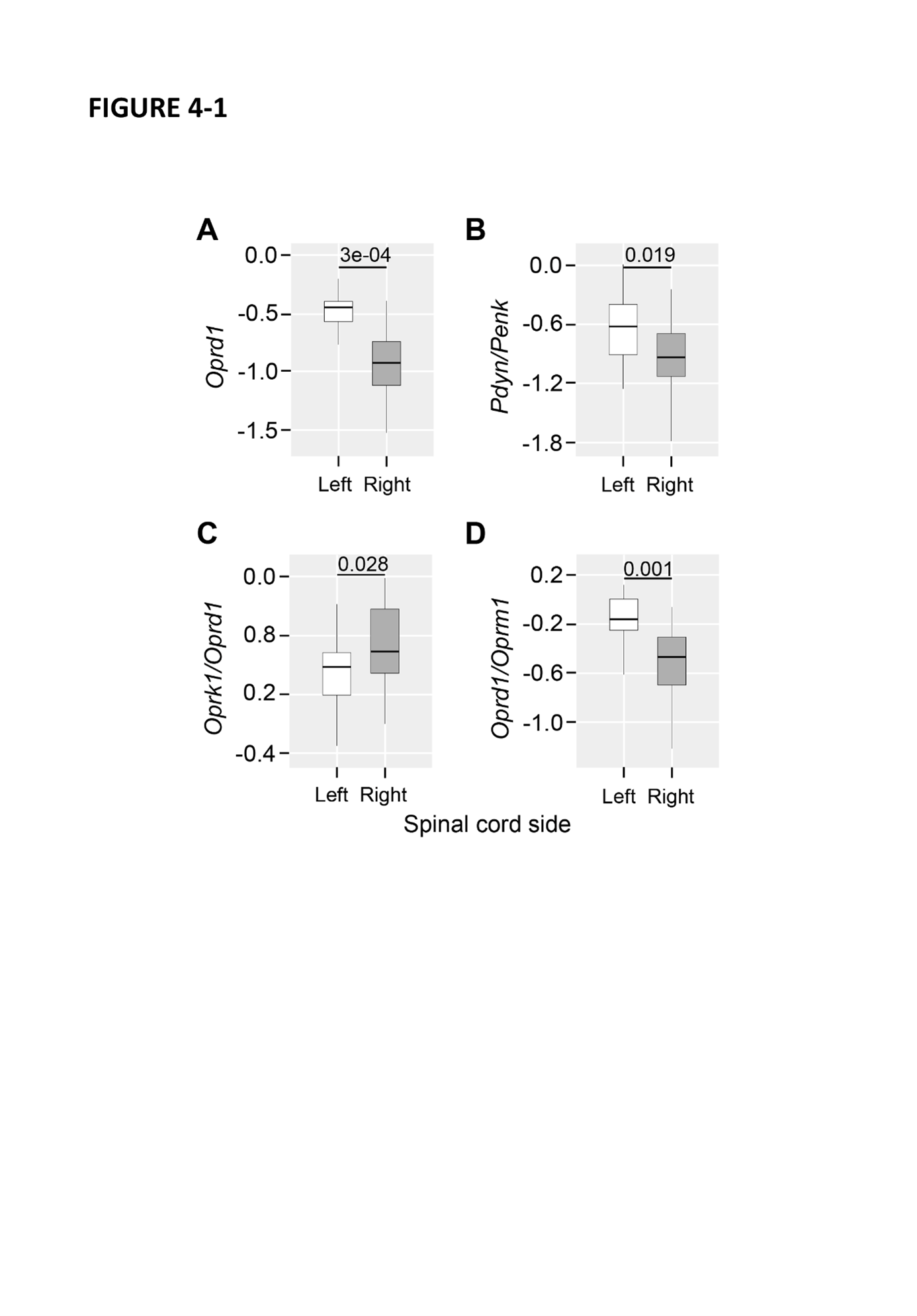

Supplement: Extended Data Figure 4-1 — Analysis of replication sample. Lateralized expression of the opioid genes in the lumbar spinal cord. Data for two animal groups (the left side sham and UBI rats; no differences between the groups in the opioid gene expression were revealed) were combined separately for each the left and right lumbar spinal halves (n = 23), Log2-scaled and compared using Wilcoxon matched pairs test. White and dark grey boxes denote the left and right spinal cords. The horizontal line in the box represents the median; the box hinges represent the first (Q1) and third (Q3) quartiles. Upper and lower whiskers extend from the hinge to the highest/lowest value that lies within the 1.5 interquartile range (IQR) of the hinge. Unadjusted p values are shown. Download Figure 4-1, TIF file. [file enu-eN-NWR-0548-20-s04.tif]
